# Psychometric Validation of the Scientific Epistemic Beliefs Questionnaire Among Mexican University Students Using Item Response Theory

**DOI:** 10.3390/jintelligence14050076

**Published:** 2026-05-02

**Authors:** José Antonio Azuela, Laura Inés Ramírez-Hernández, Osvaldo Aquines-Gutiérrez, Wendy Xiomara Chavarría-Garza, Ayax Santos-Guevara, Humberto Martínez-Huerta

**Affiliations:** 1 Department of Physics and Mathematics, Universidad de Monterrey, Avenida Morones Prieto 4500, San Pedro Garza García 66238, NL, Mexico; antonio.azuela@udem.edu (J.A.A.); wendy.chavarria@udem.edu (W.X.C.-G.); ayax.santos@udem.edu (A.S.-G.); humberto.martinezhuerta@udem.edu (H.M.-H.); 2 Faculty of Education and Humanities, Universidad de Monterrey, Avenida Morones Prieto 4500, San Pedro Garza García 66238, NL, Mexico; 3 Department of Education and Psychopedagogy, Universidad de Monterrey, Avenida Morones Prieto 4500, San Pedro Garza García 66238, NL, Mexico; laura.ramirezh@udem.edu

**Keywords:** epistemic cognition, epistemic beliefs, epistemic competences, psychometrics, item response theory, gender, PISA

## Abstract

This study examines the validity of the Spanish version of the Scientific Epistemic Beliefs (SEB) Questionnaire among university students in northeastern Mexico, considering multiple sources of evidence. The SEB measures four dimensions of epistemic beliefs: Source, Certainty, Development, and Justification. Data from pilot (*n* = 150) and main (*n* = 791) samples were analyzed using Exploratory and Confirmatory Factor Analyses (EFA, CFA), Item Response Theory (IRT), and Differential Item Functioning (DIF). The results provided evidence consistent with a four-factor model, with adequate internal consistency (α = 0.85) and acceptable-to-good fit indices (CFI = 0.944, TLI = 0.936, RMSEA = 0.067, SRMR = 0.071) for a 22-item scale. IRT analyses indicated strong item discrimination, with Source and Certainty covering a broad range of the latent trait, while Development and Justification were more informative at lower to moderate levels. DIF analyses indicated negligible differences in item functioning by gender and academic semester, with minor DIF detected across faculties. Non-parametric analyses identified statistically significant but small differences, with females scoring slightly higher across all dimensions and variations also observed across academic semesters and faculties. Descriptive comparisons with published international data provide contextual evidence within a broader cross-cultural framework.

## 1. Introduction

### 1.1. Theoretical and Developmental Foundations of Epistemic Cognition

The study of epistemic cognition—understood as a set of mental processes that involve the development and application of individuals’ conceptions of knowledge and knowing—has been shaped primarily by theoretical frameworks developed in the United States, which have guided much research in the field (Hofer, 2016; Hofer & Pintrich, 1997). Perry’s (1970) Intellectual and Ethical Development, a Piagetian stage model, provides a foundational trajectory, describing a progression from Dualism to Commitment within Relativism, in which learners move from perceiving knowledge as absolute and unchanging to recognizing multiple perspectives and making reasoned, context-sensitive commitments (Moore, 2002).

Building on this foundation, subsequent models elaborate complementary dimensions of this progression, each accentuating different aspects of epistemic growth. Belenky et al.’s (1986) Women’s Ways of Knowing highlights the evolving agency of the knower, from passive reception to constructed knowing (Clinchy, 2002). Kuhn’s (1991) Argumentative Reasoning Model emphasizes the development of reasoning from uncritical acceptance to evaluative judgment (Kuhn & Weinstock, 2002). Baxter Magolda’s (1992) Epistemological Reflection Model focuses on shifts from absolute to contextual knowing (Baxter Magolda, 2002), and King and Kitchener’s (1994) Reflective Judgment Model highlights increasingly sophisticated, evidence-based justification (King & Kitchener, 2002). Together, these models outline a shared developmental trajectory, offering nuanced perspectives on the nature of knowledge, the role of the knower, and the processes of justification, though their primarily U.S.-based origins raise questions about cross-cultural applicability.

Drawing on these developmental perspectives, Kitchener (1983) introduced the concept of epistemic cognition, defined as a higher-order process involved in reasoning about how knowledge is constructed, evaluated, and justified. Kitchener’s three-level model of cognitive processing distinguishes Level 1 (Cognition), which includes basic operations such as calculation, memorization, reading, language acquisition, perception, and problem-solving; Level 2 (Metacognition), involving awareness, planning, monitoring, regulation, and evaluation of cognitive processes; and Level 3 (Epistemic Cognition), focusing on reasoning about the justification, certainty, and boundaries of knowledge.

Unlike traditional interpretations of metacognition, which focus on monitoring and controlling cognitive processes, epistemic cognition is particularly critical when individuals encounter ill-structured problems—situations characterized by ambiguity, incomplete information, or competing evidence—requiring evaluation of information, consideration of alternative perspectives, and justification of conclusions. This positions epistemic cognition as a meta-metacognitive capacity that underlies sophisticated reasoning and informed decision-making in both educational and everyday contexts.

### 1.2. From Developmental Models to Multidimensional and Context-Sensitive Measurement

General developmental (qualitative) models of epistemic cognition have progressively given way to quantitative approaches. While developmental frameworks conceptualize epistemic beliefs as evolving through successive stages over time, quantitative models aim to assess individuals’ beliefs at a given moment, with increasing attention to context and a gradual shift from general to more domain-oriented measurement.

The quantitative, multidimensional study of epistemic cognition among students emerged with the work of Schommer (1990, 1994), who developed the Epistemological Beliefs Questionnaire (EQ), a 63-item self-report instrument based on a 5-point Likert scale. Schommer initially proposed a five-dimensional framework of epistemic beliefs encompassing Omniscient Authority, Certain Knowledge, Simple Knowledge, Quick Learning, and Fixed Ability. However, her exploratory factor analyses identified only four stable factors. The EQ nonetheless represented an important starting point for the quantitative examination of the relationships between epistemic beliefs and students’ comprehension, interpretation of information, and learning behaviors, influencing subsequent research in the field (Castañeda & Peñalosa, 2010; Chan, 2008; Elder, 2002; Schraw et al., 2002; Wood & Kardash, 2002).

Despite its widespread use, several criticisms emerged regarding its conceptual and methodological underpinnings (Buehl, 2008; Clarebout et al., 2001; Sandoval et al., 2016; Schommer-Aikins, 2004). Hofer and Pintrich (1997) noted that the exploratory factor analysis conducted in the development of the EQ did not incorporate all items from the original instrument. They also suggested that some dimensions—particularly those concerning quick learning and innate ability—were more closely related to conceptions of learning processes than to beliefs about the nature of knowledge. In response, they proposed the Personal Epistemology Model, consisting of two main constructs—the Nature of Knowledge and the Nature of Knowing—each encompassing two dimensions: Certainty and Development for the former, and Source and Justification for the latter.

Building on this framework, Conley et al. (2004) developed the Scientific Epistemic Beliefs Questionnaire (SEB), a four-dimensional instrument designed to assess students’ beliefs about scientific knowledge and knowing. While not tied to a single scientific discipline, the SEB represents a shift from general epistemic beliefs toward more context-sensitive measurement within the broader domain of science. The Source dimension reflects the belief that scientific knowledge comes from external authorities—scientists, teachers, and textbooks—whose claims are accepted without critical evaluation. Certainty captures an absolutist view of science, in which knowledge is seen as unitary, correct, and infallible. Development assesses beliefs that scientific knowledge is evolving and subject to revision. Finally, Justification focuses on the role of experimentation and inquiry as central processes for evaluating and supporting scientific claims.

### 1.3. Epistemic Cognition and Scientific Literacy in the Mexican Context

Research on epistemic cognition has important educational implications. Numerous studies have demonstrated relationships between epistemic beliefs and academic achievement (Cartiff et al., 2021; Greene et al., 2018; Guo et al., 2022), science performance (Chai et al., 2021; Wan et al., 2025; Xiong et al., 2025), and science literacy (She et al., 2019). Additionally, epistemological beliefs have been linked to science identity (Tan et al., 2024, Ulu & Yerdelen-Damar, 2024), highlighting their relevance to student engagement and long-term educational trajectories.

The significance of epistemic cognition is also reflected in international assessments such as the Organisation for Economic Co-operation and Development’s (OECD) Programme for International Student Assessment (PISA). In the 2015 science framework, PISA defined scientific literacy as encompassing three forms of knowledge: content, procedural, and epistemic (Domènech-Casal & Marbà-Tallada, 2022; Organisation for Economic Co-operation and Development [OECD], 2016, 2023b). Epistemic knowledge has since been incorporated as a central component of scientific proficiency, developing progressively across proficiency levels. At lower levels, students identify simple patterns, causal relationships, and investigable questions. At intermediate levels, they distinguish scientific from non-scientific claims, interpret data, and justify experimental designs. At the highest levels, they critically evaluate evidence, manage uncertainty, integrate multiple concepts, and reason about novel phenomena.

Regarding Mexico’s performance on PISA, science outcomes consistently lag behind the OECD average and show limited progress over time. In 2022, only about 49% of Mexican students demonstrated basic scientific literacy, compared to 76% across the OECD (Organisation for Economic Co-operation and Development [OECD], 2023c). At the upper end, high performance remains rare, with only a very small share of students reaching advanced levels, well below the OECD average of 7%. Consequently, more than half of Mexican students lack foundational scientific understanding, struggling to interpret simple explanations or assess straightforward evidence. The persistent gap in scientific literacy points to systemic barriers that may hinder the development of scientific reasoning and problem-solving skills. In this context, understanding how students conceptualize knowledge and knowing becomes particularly relevant, as epistemic beliefs may play a central role in shaping these outcomes. However, empirical research on epistemic cognition in the Mexican context remains limited, underscoring the need for further investigation.

### 1.4. Cross-Cultural Considerations in the Measurement of Epistemic Cognition

Although several instruments have been developed to assess epistemic cognition, typically operationalized through epistemic beliefs, most were created and validated in English-speaking contexts, limiting their applicability across languages and cultures. While Schommer’s EQ has been translated into Spanish (Cano, 2005; Schommer-Aikins et al., 2012), the SEB Questionnaire—despite being more widely used (S. W.-Y. Lee et al., 2021) and exhibiting robust empirical associations with academic outcomes (Greene et al., 2018)—has not yet been translated and psychometrically examined using contemporary methods, such as Item Response Theory (IRT).

Translation and cultural adaptation are critical not only for achieving linguistic equivalence but also for ensuring that instruments accurately capture epistemic cognition across diverse cultural contexts (Buehl, 2008). Providing evidence of the psychometric properties of scores derived from the Spanish version of the measure would facilitate cross-cultural comparisons, enable integration with international assessment data such as PISA, and support research on epistemic beliefs and related educational variables in Spanish-speaking contexts.

Moreover, validated instruments offer a solid foundation for evidence-based policy, curriculum design, and teacher training, thereby promoting the cultivation of 21st-century higher-order thinking skills, including epistemic competences such as reasoning about knowledge, evaluating evidence, and constructing justified understandings (Organisation for Economic Co-operation and Development [OECD], 2023b). Examining epistemic cognition across diverse educational settings advances global theories of knowledge development by integrating perspectives from multiple cultural and linguistic backgrounds, thereby broadening the field beyond its predominantly Anglo-Saxon foundations (Chan, 2008; Hofer, 2008, 2016; W. W. S. Lee & Chan, 2015; Tabak & Weinstock, 2008).

### 1.5. Study Objectives

This observational study examines evidence of the psychometric properties of scores obtained from the Spanish version of the SEB Questionnaire in a university sample from northeastern Mexico. Beyond its psychometric validation, this study examines variation in epistemic beliefs across demographic and academic factors and situates the findings within the international literature.

This study is addressed through three research questions:

- (RQ1) Does the Spanish SEB Questionnaire exhibit adequate psychometric properties in Mexican university students?
- (RQ2) Do epistemic beliefs differ by gender, academic semester, and faculty?
- (RQ3) How do Mexican students’ epistemic belief scores compare with international findings across educational levels?

## 2. Materials and Methods

### 2.1. Research Design

This study adopts a quantitative design to examine the psychometric properties of the SEB Questionnaire scores. Cross-sectional data from university students at a private institution in northeastern Mexico were analyzed through Exploratory Factor Analysis (EFA), internal consistency estimates, Confirmatory Factor Analysis (CFA), and Item Response Theory (IRT) modeling, including Item Information Curves (IICs), and Test Characteristic Curves (TCCs). Item-level invariance was examined using Differential Item Functioning (DIF), and group differences were analyzed using nonparametric tests.

### 2.2. Participants

#### 2.2.1. Pilot Sample (Fall 2024)

Prior to the main study, an independent sample of 150 students from the selected private university completed the questionnaire. This sample was used exclusively for EFA to examine the instrument’s underlying factor structure. The sample included 81 female students (54.0%), 66 male students (44.0%), and 3 students identifying as diverse (2.0%). Participants were enrolled across five faculties: Art, Architecture, and Design (AAYD; 13 students, 8.7%), Health Sciences (CS; 17, 11.3%), Education and Humanities (EYH; 18, 12.0%), Engineering and Technologies (IYT; 39, 26.0%), and Business (N; 63, 42.0%). Students were distributed across semesters 1 to 9, corresponding to an age range of 18 to 22 years, with the largest groups in semesters 1 (61.3%), 5 (11.3%), 7 (10.0%), and 9 (7.3%).

#### 2.2.2. Main Sample (Spring 2025)

The main sample consisted of 791 Mexican students from the selected private university who completed the instrument for CFA and subsequent analyses. Regarding gender, 436 students (55.1%) identified as female, and 355 students (44.9%) as male. Students were enrolled across six faculties, as shown in Table 1: AAYD, CS, Law and Social Sciences (DYCS), EYH, IYT, and N. Semester distribution ranged from 1 to 10, corresponding to ages 18 to 23 years, with the largest groups in semesters 2 (21.4%), 4 (29.2%), 6 (22.8%), and 8 (11.3%).

### 2.3. Instrument

The 26 items of the SEB Questionnaire (Conley et al., 2004) were translated into Spanish by a bilingual translator and reviewed by a team of experts in science education to ensure linguistic clarity, cultural appropriateness, and preservation of the original meaning for Mexican university students (see Appendix A). The translated items were subsequently reviewed in the Fall 2024 pilot sample used for EFA and were considered appropriate for the study. Permission for reproduction and translation was obtained from the copyright holder, Elsevier.

SEB items are rated on a 5-point Likert scale (1 = strongly disagree, 5 = strongly agree) and are designed to focus on students’ beliefs in the domain of science. Items for the Source and Certainty dimensions were reverse-coded during data collection, following the original scale’s instructions; however, for consistency across the entire instrument during analysis, these scores were recoded back to their intended response direction before performing the factor analyses.

The full item set consists of:

**Source**

1. Everybody has to believe what scientists say.
2. In science, you have to believe what the science books say about stuff.
3. Whatever the teacher says in science class is true.
4. If you read something in a science book, you can be sure it’s true.
5. Only scientists know for sure what is true in science.

**Certainty**

6. All questions in science have one right answer.
7. The most important part of doing science is coming up with the right answer.
8. Scientists pretty much know everything about science; there is not much more to know.
9. Scientific knowledge is always true.
10. Once scientists have a result from an experiment, that is the only answer.
11. Scientists always agree about what is true in science.

**Development**

12. Some ideas in science today are different than what scientists used to think.
13. The ideas in science books sometimes change.
14. There are some questions that even scientists cannot answer.
15. Ideas in science sometimes change.
16. New discoveries can change what scientists think is true.
17. Sometimes scientists change their minds about what is true in science.

**Justification**

18. Ideas about science experiments come from being curious and thinking about how things work.
19. In science, there can be more than one way for scientists to test their ideas.
20. One important part of science is doing experiments to come up with new ideas about how things work.
21. It is good to try experiments more than once to make sure of your findings.
22. Good ideas in science can come from anybody, not just from scientists.
23. A good way to know if something is true is to do an experiment.
24. Good answers are based on evidence from many different experiments.
25. Ideas in science can come from your own questions and experiments.
26. It is good to have an idea before you start an experiment.

### 2.4. Application Procedure

Data were collected from two samples of students enrolled in the Scientific and Technological Thinking course at the selected university: a pilot sample of 150 students in Fall 2024 and a main sample of 791 students in Spring 2025. Surveys were administered in class via Google Forms under instructor supervision. Participation was voluntary, informed consent was obtained from all students, and anonymity was maintained throughout the research process. Administration procedures and confidentiality measures were identical for both samples.

### 2.5. Statistical Analysis and Data Processing

To address RQ1, an initial EFA was conducted on the pilot sample (Fall 2024) to examine the factor structure of scores. A refinement EFA was subsequently conducted on the main sample (Spring 2025) to verify the factor structure identified in the pilot phase and to remove items exhibiting cross-loadings. This sequential approach ensured initial exploration followed by verification and refinement in a larger, independent sample, thereby enhancing the stability and generalizability of the measurement model.

In both analyses, internal consistency was assessed using Cronbach’s alpha coefficient (Cronbach, 1951). According to conventional standards, values of α < 0.60 indicate unacceptable reliability, α = 0.60–0.69 suggest marginal reliability, α = 0.70–0.79 are considered acceptable, α = 0.80–0.90 indicate good reliability, and values of α > 0.90 reflect excellent reliability (Cohen et al., 2018). All EFA procedures were conducted using IBM SPSS Statistics (Version 30; IBM Corp., 2024).

To evaluate measurement equivalence across groups, both CFA and IRT were employed. CFA provides evidence of the structural comparability of latent constructs, whereas IRT focuses on item-level invariance and offers greater sensitivity for detecting specific item bias. This combined approach provides multiple sources of evidence relevant to the interpretation of scores by integrating complementary perspectives (Tay et al., 2015), thereby strengthening both structural support and item-level precision.

CFA was performed using the Weighted Least Squares Mean and Variance adjusted (WLSMV) estimator, suitable for ordinal indicators, with all items treated as ordered variables. CFA model fit was evaluated using standard indices, including the Root Mean Square Error of Approximation (RMSEA), the Tucker–Lewis Index (TLI), the Standardized Root Mean Square Residual (SRMR), and the Comparative Fit Index (CFI), with acceptable model fit defined as RMSEA < 0.07, SRMR ≤ 0.08, and TLI and CFI > 0.92 (Hair et al., 2019). These thresholds were selected based on widely accepted guidelines in psychometric research to ensure rigorous evaluation of model fit.

To evaluate the internal consistency and gather evidence supporting convergent–discriminant relations among factors, Composite Reliability (CR), Average Variance Extracted (AVE), and Maximum Shared Variance (MSV) were calculated based on the standardized factor loadings obtained from CFA. CR was used to assess internal consistency, with values ≥ 0.70 considered acceptable (Cohen et al., 2018). Evidence of convergent validity was assessed by calculating the AVE, with a minimum threshold of 0.50 required to indicate that a factor explains more than half of the variance of its corresponding items. Discriminant validity was established using two criteria. First, the Fornell and Larcker (1981) criterion was applied, which requires the √AVE for each latent factor to be greater than its highest standardized inter-factor correlation (r). Second, discriminant validity was further supported by evidence that the AVE for each construct exceeded its MSV.

Prior to conducting the IRT analyses, items exhibiting cross-loadings in the main EFA or high residual errors in the CFA were removed. Unidimensionality within each factor was then assessed using residual-based diagnostics derived from the CFA model. Residual correlations were examined to assess potential local dependence, with values exceeding |0.20| and proportions above approximately 5% considered indicative of potential violations (Finch & Jeffers, 2016).

IRT analyses were performed using the Graded Response Model (GRM; Samejima, 1969), suitable for ordered polytomous data such as 5-point Likert scales. The GRM estimates item parameters—discrimination (a) and thresholds (b_1_–b_4_)—providing sample-independent metrics to evaluate item quality. Discrimination values are classified as moderate (0.65–1.34), high (1.35–1.69), and very high (>1.69), reflecting an item’s ability to distinguish respondents across the latent trait continuum (θ), examined between −3 and +3 to provide information across levels of the latent trait (Baker, 2001; Toland, 2013). Item Information Curves (IICs) were visually inspected to assess each item’s precision.

DIF analyses were subsequently performed to detect items exhibiting differential functioning across gender, academic semester, and faculty groups, as such disparities could indicate potential sources of bias affecting score interpretations across groups. DIF detection employed Likelihood Ratio χ^2^ tests comparing nested models: Model 1 vs. Model 2 (χ^2^_12_) to identify uniform DIF, Model 1 vs. Model 3 (χ^2^_13_) for non-uniform DIF, and Model 2 vs. Model 3 (χ^2^_23_), for global DIF. Uniform DIF indicates a consistent advantage for one group across all levels of the latent trait (θ), typically represented by parallel Item Characteristic Curves (ICCs). In contrast, non-uniform DIF reflects variation in group advantage across trait levels, manifested as diverging or crossing ICCs.

To evaluate the magnitude of DIF, multiple effect size indicators were computed. These included changes in McFadden’s pseudo-*R*^2^ and Nagelkerke’s pseudo-*R*^2^ between models, as well as β_12_, defined as the absolute proportional change in the slope parameter (a) for the latent trait when comparing Models 1 and 2. Following commonly adopted criteria for interpreting the Likelihood Ratio Tests (LRTs), a significance level of α < 0.05 was used alongside effect size thresholds (pseudo-*R*^2^ changes > 0.02 and β_12_ > 0.03) to determine whether DIF was substantively meaningful (Choi et al., 2011). Items were flagged for DIF only if both significance and effect size criteria were met.

To address RQ2, comparisons among three or more groups were performed using the Kruskal–Wallis test, with effect sizes quantified using eta squared (η^2^_H). For pairwise comparisons between two groups—such as between gender and specific academic semesters or faculties—the Mann–Whitney U test was applied, with effect sizes calculated using the correlation coefficient (r). This approach enables accurate detection of significant differences and appropriate measurement of effect sizes across multiple groups.

Finally, a descriptive comparative analysis was conducted to address RQ3. The results were contextualized by contrasting mean scores and standard deviations with published international findings across different educational levels and cultural contexts. This qualitative descriptive comparison situates the epistemic profiles of Mexican students within the broader body of global empirical evidence.

All advanced analyses were conducted in R (Version 4.5.1; R Core Team, 2025). CFA was carried out using *lavaan* (v0.6.20; Rosseel, 2012) with the WLSMV estimator, and model visualization employed *semPlot* (v1.1.7; Epskamp, 2019). The GRM, IICs, ICCs and TCCs were obtained using *mirt* (v1.45.1; Chalmers, 2012) and visualized with *ggplot2* (v4.0.0; Wickham, 2016). DIF analyses were conducted using *lordif* (v0.4.2; Choi et al., 2011).

## 3. Results

### 3.1. Initial EFA (Pilot Sample, Fall 2024)

A preliminary EFA was conducted with a sample of 150 university students using principal component analysis with Varimax rotation and Kaiser normalization. The rotation converged in seven iterations. The suitability of the data for factor analysis was confirmed by a Kaiser–Meyer–Olkin (KMO) measure of sampling adequacy of 0.867, indicating meritorious sampling adequacy. Bartlett’s test of sphericity was significant, χ^2^(325) = 2129.55, *p* < 0.001, supporting the factorability of the correlation matrix.

Reliability analysis of the 26-item instrument indicated strong internal consistency (Cronbach’s alpha = 0.89), suggesting coherent measurement of the construct. The rotated factor loadings supported the hypothesized four-dimensional structure of the scale. Most items loaded strongly on their expected factors (>0.55), though some showed moderate cross-loadings between Source and Certainty (item 3), and between Development and Justification (items 14, 15, and 16), indicating partial overlap (see Appendix B for full factor loadings).

### 3.2. Refinement EFA and Reliability (Main Sample, Spring 2025)

**Figure 1:**
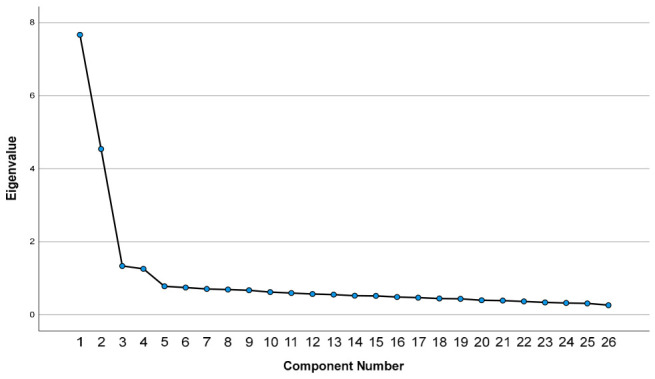
Scree plot displaying the eigenvalues of extracted components from the 26-item SEB Questionnaire, supporting the retention of four factors.

Prior to conducting confirmatory analyses, a refinement EFA was performed on the main sample of 791 university students to verify the factor structure identified in the pilot phase and to address items exhibiting cross-loadings. The adequacy of this sample for factor analysis was assessed using the KMO measure and Bartlett’s test of sphericity. The KMO value was 0.935, indicating excellent sampling adequacy and suggesting that the correlations among items were suitable for factor extraction. Bartlett’s test of sphericity was significant, χ^2^(325) = 9174.39, *p* < 0.001. The Justification factor explained 18.76% of the variance; Development accounted for 15.20%; Certainty explained 13.61%; and Source contributed 9.36%. Components beyond the fourth contributed minimally and were excluded from interpretation. The adequacy of this four-factor structure was indicated by both the scree plot (see Figure 1) and Kaiser’s criterion (eigenvalues > 1).

These results support a four-component solution as a meaningful and interpretable representation of the data’s underlying structure. Factor loadings of ≥0.40 were considered significant for interpretation (see Table 2). However, unlike the original SEB factor structure proposed by Conley et al. (2004), item 5 *(“Only scientists know for sure what is true in science.”)*, loaded more strongly on the Certainty factor than on Source in this sample. Additionally, item 16 *(“New discoveries can change what scientists think is true.”)*, item 18 *(“Ideas about science experiments come from being curious and thinking about how things work.”)*, and item 19 *(“In science, there can be more than one way for scientists to test their ideas.”)*, exhibited the highest cross-loadings between Development and Justification. These items were excluded from further analysis due to insufficient differentiation, which compromised factorial purity and interpretability; their removal improved the conceptual distinctiveness of the factors and the clarity of the solution.

After deriving the final factor structure from the EFA on the main sample (*n* = 791), which involved the elimination of items 16, 17, and 18, the internal consistency of each factor was assessed using Cronbach’s alpha coefficients for the resulting 23-item model. As presented in Table 3, all subscales exceeded the recommended *α* threshold of 0.70, and coefficients ranged from 0.766 to 0.896. The Justification (α = 0.896), Development (α = 0.868), and Certainty (α = 0.827) subscales, along with the Total SEB scale (α = 0.868), exhibited strong internal consistency, while the Source subscale presented acceptable consistency (α = 0.766), supporting the reliability of the instrument’s dimensions in this sample.

### 3.3. Confirmatory Factor Analysis and Construct Validity

A CFA was initially conducted on the 23-item version (see Appendix C). However, item 7 *(“The most important part of doing science is coming up with the right answer.”)*, exhibited a relatively low factor loading of 0.46 and a high residual error of 0.79, indicating that a substantial portion of its variance was unexplained by the factor. Consequently, item 7 was removed in a subsequent model to improve parameter estimation and model fit, thereby ensuring more reliable estimation of the latent constructs.

The overall fit of the 22-item model was slightly superior to that of the 23-item model. Although both models demonstrated acceptable goodness-of-fit values according to conventional criteria, the 22-item version achieved marginally better fit indices (CFI = 0.944, TLI = 0.936, RMSEA = 0.067, SRMR = 0.071) compared to the 23-item model (CFI = 0.939, TLI = 0.933, RMSEA = 0.067, SRMR = 0.075).

**Figure 2:**
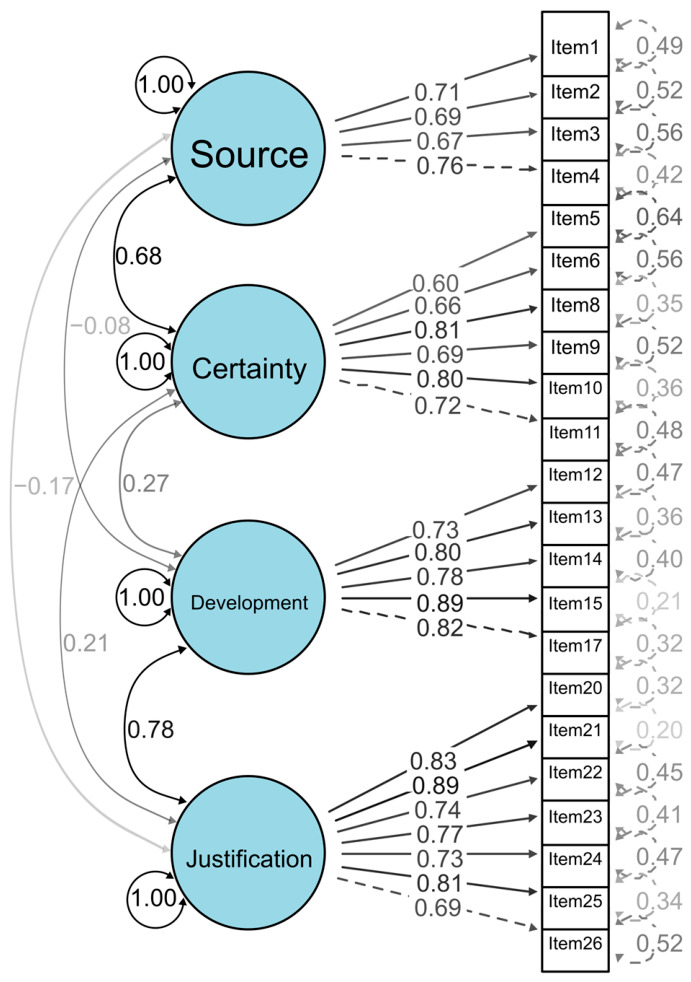
Confirmatory Factor Analysis (CFA) model of the 22-item SEB Questionnaire.

As shown in Figure 2, all standardized factor loadings ranged from 0.60 to 0.90 (*p* < 0.001), indicating adequate to strong item–factor associations. The Source dimension exhibited loadings between 0.67 and 0.76, reflecting adequate representation of the construct. The Certainty dimension showed more variability, with loadings from 0.60 to 0.81, indicating moderate to strong item–factor relations. The Development and Justification dimensions showed the highest loadings, with most items exceeding 0.70 and reaching up to 0.89, suggesting strong associations with their respective constructs following the removal of items 16, 18, and 19.

Regarding error variances, 21 of the 22 items showed values between 0.20 and 0.56, indicating acceptable levels of unexplained variance. Only item 5 showed a relatively higher error variance (0.64) suggesting lower measurement precision compared to the remaining items. Overall, the CFA results provide evidence consistent with the proposed factor structure of the scale.

As detailed in Table 4, the latent factor variances (*ψ*) were all statistically significant (*p* < 0.001), indicating meaningful variability in participant responses across the four factors. The Development factor exhibited the highest variance (*ψ* = 0.675, SE = 0.032) reflecting greater heterogeneity among respondents. In contrast, the Justification factor showed the lowest variance (*ψ* = 0.483, SE = 0.030), suggesting more homogeneity in responses.

Table 5 presents reliability estimates and evidence related to convergent and discriminant validity. CR values (0.80–0.92) indicate good internal consistency across the four constructs, while AVE values (0.50–0.65) provide evidence supporting convergent validity. For Source, Certainty, and Development, AVE values were higher than MSV, which is consistent with adequate separation between constructs. In contrast, for Justification, AVE and MSV were very similar, suggesting less clear separation from the other constructs.

According to the Fornell–Larcker criterion, evidence regarding discriminant validity was generally acceptable, as the square roots of the AVEs exceeded the inter-factor correlations for most constructs. Source and Certainty exhibited low to moderate correlations with other factors (|r| = 0.084–0.685), indicating satisfactory factorial distinctiveness. In contrast, Development and Justification showed a relatively high correlation (r = 0.780), approaching the square roots of their AVEs (√AVE = 0.804 and 0.784), suggesting substantial shared variance and some degree of conceptual overlap. Taken together, these findings are consistent with the decision to remove items 16, 18, and 19.

### 3.4. Item Response Theory Analysis

#### 3.4.1. Discrimination (a), Threshold (b) Parameters, and Item Information Curves (IICs)

Prior to conducting IRT analyses, the unidimensionality of items within each factor was evaluated through residual analysis. The results indicated that the vast majority of residuals were small, with only 1.73% exceeding |0.20|. This pattern suggests that the model adequately reproduces the observed correlations and that no substantial violations of local independence are present.

The GRM analysis indicated that the items across the four factors showed adequate to strong psychometric functioning. As shown in Table 6, discrimination parameters (a) ranged from 1.32 (item 5) to 4.30 (item 15), suggesting that the items were generally effective in differentiating respondents along their respective latent dimensions.

The Source and Certainty items displayed thresholds (b) covering a wide range of the latent continuum (from −2.58 to +2.91), indicating that these items provide information across low to high levels of epistemic beliefs. Conversely, Development and Justification items exhibited predominantly negative threshold values (−4.51 to −0.04), implying that they are most informative for respondents located at lower and moderate levels of the latent trait. Overall, the discrimination and threshold parameters suggest that the scale captures variation in epistemic beliefs across its four dimensions.

**Figure 3:**
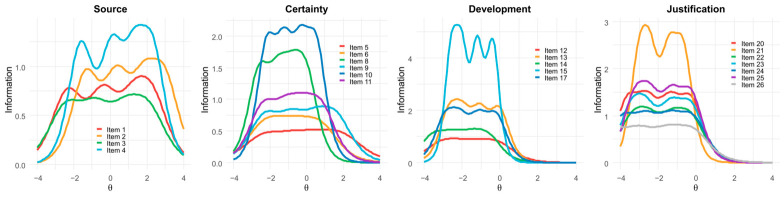
Item Information Curves (IICs) for the 22-item model of the SEB Questionnaire.

Figure 3 displays the Item Information Curves (IICs). The most informative items for each factor were item 4 (Source), item 10 (Certainty), item 15 (Development), and item 21 (Justification), whereas items 5 (Certainty), 12 (Development), and 26 (Justification) contributed the least information.

#### 3.4.2. Differential Item Functioning (DIF) Analysis

Table 7 presents the gender-based DIF analysis conducted using Likelihood Ratio χ^2^ Tests (LRT) to evaluate potential bias in the 22-item SEB Questionnaire. The table reports *p*-values and corresponding degrees of freedom for uniform (χ^2^_12_), non-uniform (χ^2^_13_), and global (χ^2^_23_) DIF, along with effect size indicators—McFadden’s and Nagelkerke’s pseudo-*R*^2^—and the proportional change in the discrimination parameter (β_12_).

As shown, most items presented *p* > 0.05, indicating no statistically significant DIF. Only Items 4, 6, and 21 yielded *p* < 0.05 in at least one comparison, suggesting possible statistical DIF. However, all effect sizes were negligible: pseudo-*R*^2^ changes remained below 0.02, and β_12_ values did not reach the recommended threshold for substantive DIF (β_12_ > 0.03). Accordingly, even items with significant *p*-values demonstrated trivial practical impact.

**Figure 4:**
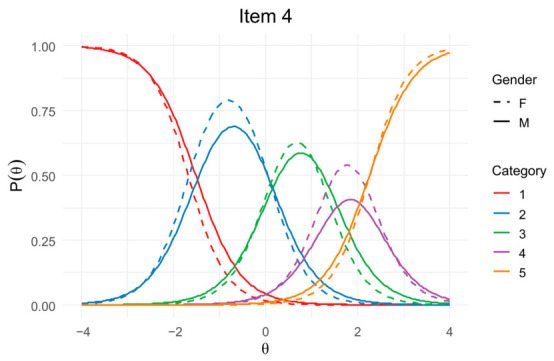
Item Characteristic Curves (ICCs) by gender for item 4.

Figure 4 displays the Item Characteristic Curves (ICCs) for item 4 *(“If you read something in a science book, you can be sure it’s true.”)*, illustrating the response probabilities of female and male respondents across the latent trait continuum (θ). Visual inspection points to differences between groups consistent with both uniform and non-uniform DIF; however, statistical tests indicate that these differences, although significant (χ^2^_12_ and χ^2^_13_: *p* = 0.003), are negligible (pseudo-*R*^2^ ≤ 0.006, β_12_ ≤ 0.019), suggesting no meaningful evidence of gender bias in item category endorsement.

**Figure 5:**
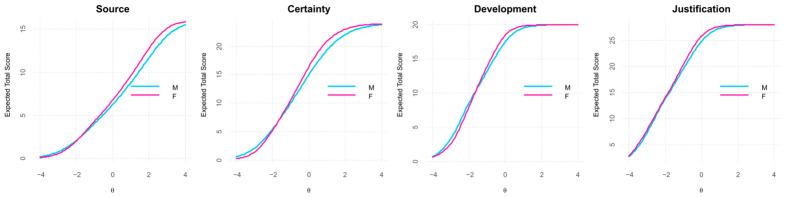
Test Characteristic Curves (TCCs) by gender for the 22-item SEB Questionnaire.

As shown in Figure 5, the influence of item-level differences on the Test Characteristic Curves (TCCs) was minimal. Overall, the gender groups exhibited greater similarity on the Development and Justification dimensions than on Source and Certainty. At moderate to high levels of the latent trait, females displayed slightly higher expected scores, whereas at low to moderate levels the curves largely overlapped. Importantly, given that the DIF analysis did not indicate significant measurement bias, these differences are likely to reflect genuine variation in the underlying trait rather than artifacts of differential item functioning. A more detailed comparison between groups is presented in a later section.

Regarding DIF across academic semesters, even-numbered semesters (2, 4, 6, and 8) were deliberately selected due to their higher student enrollment during the spring term, representing 84.7% of the study sample (*n* = 669). As presented in Appendix D.1, most items displayed non-significant DIF effects, with *p*-values > 0.05 across uniform, non-uniform, and global tests, suggesting no meaningful evidence of bias associated with semester. Although items 13 and 26 yielded the lowest *p*-values in some comparisons (0.0002 and 0.014, respectively), these did not reflect a consistent pattern of statistical significance. McFadden’s and Nagelkerke’s pseudo-*R*^2^ values were uniformly minimal (<0.02), and β_12_ estimates did not exceed the 0.03 threshold, further supporting a minimal practical impact of DIF.

**Figure 6:**
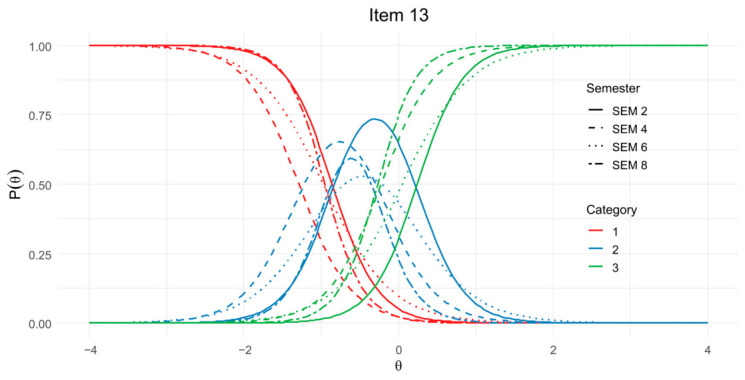
Item Characteristic Curves (ICCs) for item 13 by academic semester.

Figure 6 displays the ICCs for item 13 *(“The ideas in science books sometimes change.”)*, disaggregated by academic semester. Given the sparse use of the lower response options—only 3.6% of responses corresponded to categories 1 and 2—the Likert scale categories 1–3 were collapsed into a single category to enable clearer visual comparison. The ICCs show that, across semesters, response probabilities follow a consistent pattern: Category 1 is most probable at low levels of the latent trait (θ), Category 2 peaks around the midpoint, and Category 3 becomes more likely at higher θ values, consistent with expectations for graded response items. While the plot highlights uniform DIF differences in the extreme categories and non-uniform DIF in the intermediate categories, the effect size was not large enough to flag any item.

A noteworthy observation is that item 13, like item 4, refers specifically to science books, and both items emerge as the most unstable or “noisy” in the DIF analyses by academic semester and gender, respectively. This convergence suggests that item content related to textbooks may be particularly sensitive to subgroup differences in interpretation or response behavior.

**Figure 7:**
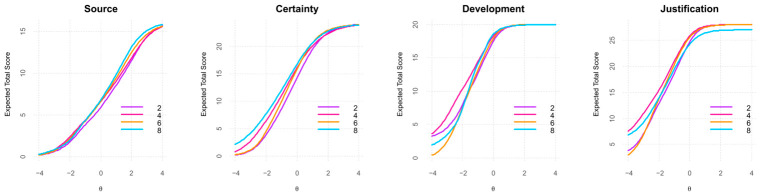
Test Characteristic Curves (TCCs) by academic semester for the 22-item SEB Questionnaire.

As shown in Figure 7, the uniform DIF detected at lower levels of Development and Justification appears to be driven primarily by the limited use of Likert scale options 1 and 2, with responses instead clustering around options 3 and 5. Given that no item exceeded the DIF detection thresholds, this pattern is more likely to reflect characteristics of the sample rather than systematic differences in item functioning.

Regarding the DIF analysis by faculty (see Appendix D.2), categories 1, 2, and 3 for item 21 were collapsed prior to model estimation due to low response frequencies. Overall, the results indicated that most of the 22 items did not exhibit statistically significant DIF, as reflected by non-significant *p*-values across the three LRT comparisons. Likewise, effect size indicators—McFadden’s and Nagelkerke’s pseudo-*R*^2^—remained consistently below the established 0.020 threshold for the majority of items, suggesting negligible practical impact. In contrast, items 3, 15, and 24 showed statistically significant DIF, with *p* < 0.05 and pseudo-*R*^2^ values exceeding 0.02 in several comparisons, indicating potential differential functioning across faculties.

**Figure 8:**
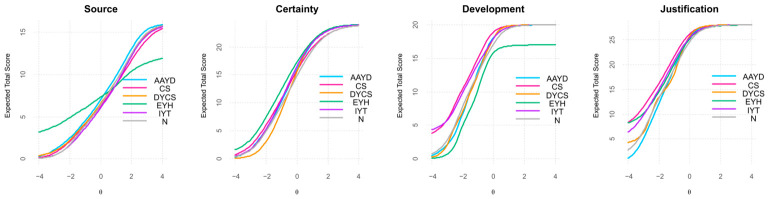
Test Characteristic Curves (TCCs) by faculty for the 22-item SEB Questionnaire.

Figure 8 presents the Test Characteristic Curves by faculty. As shown, there is a pronounced response pattern in the curves for the Source and Development dimensions among participants from the Faculty of Education and Humanities (EYH), a pattern likely attributable to this group’s underrepresentation (*n* = 47). In contrast, the Certainty and Justification dimensions display a more uniform response pattern across faculties.

**Figure 9:**
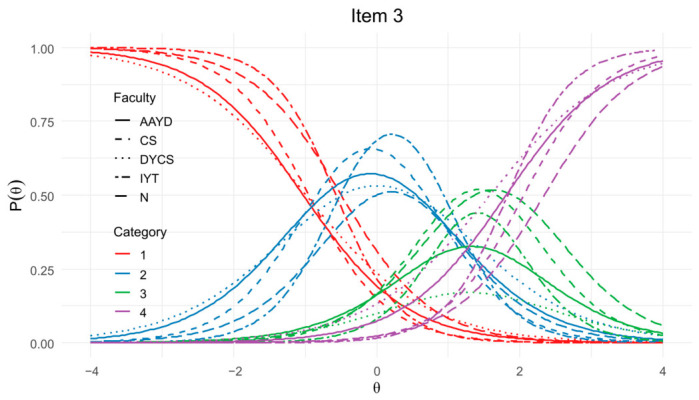
Item Characteristic Curves (ICCs) for item 3 by faculty.

Following inspection of the TCCs, a DIF model excluding the EYH faculty was estimated (*n* = 744). The results showed that items 3 and 24 continued to exhibit both uniform and non-uniform DIF; however, the pseudo-*R*^2^ values for all three flagged items remained below the 0.02 threshold, and the β_12_ coefficients were below 0.030. In light of these findings, and considering the underrepresentation of EYH, this faculty was excluded from subsequent cross-faculty analyses. Figure 9 illustrates the ICCs for item 3 *(“Whatever the teacher says in science class is true.”)*, following this adjustment.

### 3.5. Comparative Analysis of Epistemic Beliefs

#### 3.5.1. Gender Differences in Epistemic Beliefs

The Mann–Whitney U tests (Table 8) revealed statistically significant differences between genders in all dimensions of the SEB construct. Specifically, Certainty, and the Total SEB score showed the most significant group differences, all with small effect sizes (r = 0.090–0.152).

Table 9 presents the descriptive statistics of SEB scores, separated by gender. Female participants (*n* = 436) consistently exhibited slightly higher mean scores than male participants (*n* = 355) across all dimensions. This trend was also observed in the Total SEB score, with females demonstrating a higher mean (M = 3.92, SD = 0.46) compared to males (M = 3.78, SD = 0.45). The largest gender difference was found in the Certainty dimension (M = 3.64, SD = 0.84 for females vs. M = 3.45, SD = 0.81 for males).

**Figure 10:**
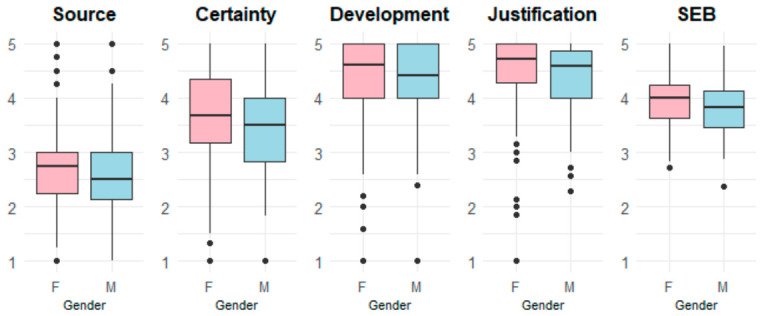
Comparison of SEB scores by gender.

Figure 10 displays boxplots illustrating the distribution of scores across the SEB dimensions by gender. The central line within each box denotes the median, while the boxes represent the interquartile range (IQR). Whiskers extend to the minimum and maximum values within 1.5 times the IQR, with outliers depicted as individual points beyond these limits. Across all dimensions, female participants demonstrate higher median scores than males. Specifically, median scores for females versus males are 2.75 vs. 2.50 for Source, 3.67 vs. 3.50 for Certainty, 4.60 vs. 4.40 for Development, and 4.71 vs. 4.57 for Justification. For the Total SEB score, female students similarly score higher (4.00 vs. 3.91).

#### 3.5.2. Differences in Epistemic Beliefs by Academic Semester

Given the prior gender-based findings, the Kruskal–Wallis analyses were conducted separately for male and female groups. Analyses of SEB dimensions across academic semesters focused on Semesters 2, 4, 6, and 8 due to higher enrollment levels (see Table 10).

For the male group, none of the SEB dimensions showed significant differences across academic semesters (α = 0.05). In contrast, for the female group, significant semester effects emerged for Source (H(3) = 13.04, *p* = 0.005, η^2^_H = 0.026), and Certainty (H(3) = 9.77, *p* = 0.021, η^2^_H = 0.018), both reflecting small effects. Total SEB also differed significantly across semesters for the female group (H(3) = 10.23, *p* = 0.017, η^2^_H = 0.019).

Given significant differences in SEB scores among females, pairwise Mann–Whitney U tests were used to identify significant semester differences. The key results are presented in Table 11. For the Source dimension, significant differences were found between semesters 2 vs. 4 (*p* = 0.027), 2 vs. 6 (*p* = 0.016), and 2 vs. 8 (*p* = 0.015). For the Certainty dimension, only the comparison between semesters 2 vs. 8 was significant (*p* = 0.043). For Total SEB, a significant difference emerged only between semesters 2 vs. 4 (*p* = 0.024). Across all analyses, significant pairwise differences were associated with small effect sizes.

As shown in Table 12, female students demonstrated gradual increases in Source, Certainty, and Total SEB scores across academic semesters. For Source, mean values rose from Semester 2 to 8 (M = 2.45–2.85). Certainty scores followed a similar upward trend, increasing from Semester 2 to 8 (M = 3.36–3.80). Total SEB scores also showed modest growth, rising from 3.76 to 4.00 across the same period. Overall, these descriptive patterns indicate slight but consistent strengthening of epistemic belief dimensions as female students advance through their academic programs.

**Figure 11:**
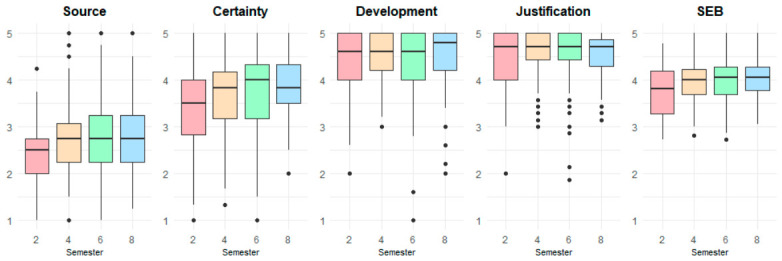
Comparison of SEB scores by academic semester for female participants.

Figure 11 shows the distribution of SEB dimensions across semesters for the female group. For the Source dimension, median values remain stable from Semester 4 to 8 (Median = 2.50 to 2.75). Certainty displayed an upward shift (Median = 3.50–4.00), with higher semesters showing more concentrated distributions around the upper Likert categories. Development (Median = 4.60–4.80) and Justification (Median = 4.71) display consistently high and stable central tendency across all semesters. Finally, Total SEB scores exhibited a gradual increase, with Semester 8 showing the highest central tendency and reduced variability. Overall, the boxplots illustrate a modest upward trend, indicating that female students tend to report more advanced epistemic beliefs as they progress through their programs.

**Figure 12:**
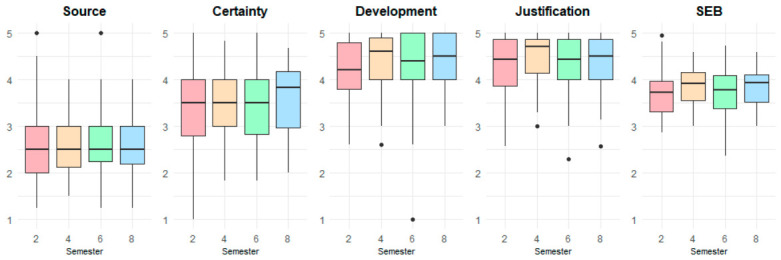
Comparison of SEB scores by academic semester for male participants.

For the male group (Figure 12), the distribution of SEB dimensions across semesters shows relatively stable patterns with modest variation. Source scores remain fairly consistent over time (Median = 2.50). Certainty exhibits a mild upward trend, suggesting somewhat greater confidence in epistemic judgments among later-semester students (Median = 3.50–3.83). Development and Justification maintain high scores across all semesters (Median = 4.20–4.71), indicating strong and stable epistemic reasoning in these dimensions.

#### 3.5.3. Differences in Epistemic Beliefs by Faculty

As noted in the DIF analysis by faculty (see Figure 8), the Education and Humanities (EYH) group was excluded from the Kruskal–Wallis analyses to prevent biased or misleading interpretations. The results for the remaining faculties are presented in Table 13.

Given the significant differences in SEB scores among faculties, pairwise comparisons were conducted using Mann–Whitney U tests to determine which faculties differed significantly. The key results are presented in Table 14.

Although the Kruskal–Wallis tests did not reveal significant overall differences for Source or Certainty, the pairwise Mann–Whitney U tests identified localized contrasts between specific faculties. For Source, AAYD and IYT differed significantly (*p* = 0.046). For Certainty, a significant difference was observed between IYT and N (*p* = 0.032). Significant contrasts also emerged between CS and N for Development (*p* < 0.001), Justification (*p* = 0.001), and Total SEB (*p* = 0.0005). All effect sizes were small, indicating that the practical impact of the differences was limited.

The comparison of mean scores across faculties reveals notable differences in the four dimensions measured (see Table 15). For Source, AAYD shows the highest mean (2.80), while IYT has the lowest (2.55). In Certainty, IYT leads with a mean of 3.68, slightly above other faculties clustered around 3.42 to 3.60. Regarding Development and Justification, CS registers the highest means (4.54 and 4.56, respectively), while N scores the lowest (4.24 and 4.33). Lastly, in Total SEB, CS presents the highest mean (3.93), closely followed by IYT (3.89), and N the lowest (3.76).

**Figure 13:**
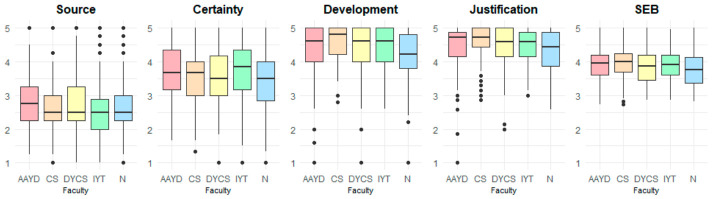
Comparison of SEB scores by faculty.

Comparing median scores across faculties (see Figure 13), most faculties show a median of 2.50 on the Source dimension. For Certainty, the IYT faculty displays a higher median (3.83) relative to the remaining faculties. Development and Justification exhibit consistently high medians across faculties (4.57–4.80), with the exception of the Business faculty (N), which shows comparatively lower medians of 4.20 and 4.43, respectively. For the Total SEB score, medians cluster within a narrow range (3.75–4.00), suggesting comparable central tendencies across faculties. Overall, these results indicate only limited variability at the faculty level.

#### 3.5.4. International SEB Cohort Comparative Analysis

**Figure 14:**
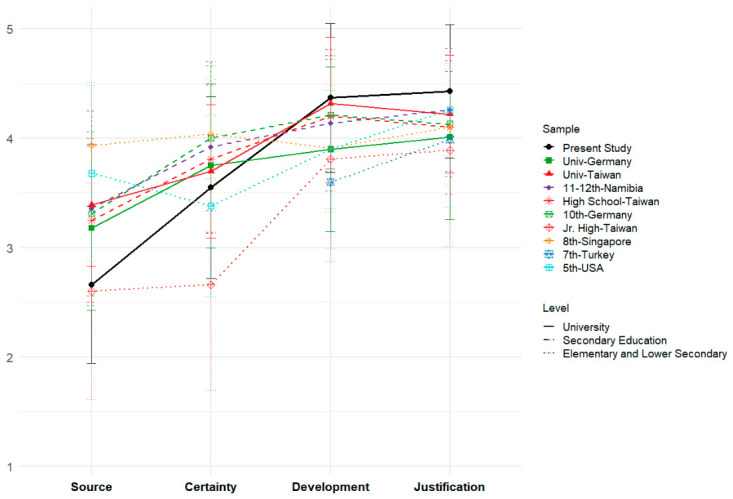
Mean SEB Scores by Dimension Across Diverse Educational Levels and Cultural Contexts.

Table 16 summarizes the mean scores and standard deviations for each SEB dimension from selected empirical studies conducted across various countries and educational levels. Figure 14 illustrates that, except for Singapore—which exhibits consistently high and stable scores across all four dimensions (ranging from 3.91 to 4.10)—a common pattern emerges across samples, with scores generally increasing sequentially from Source through Certainty, and peaking at Development and Justification.

In general, trajectories for Development and Justification show the expected increase from elementary to university levels, consistently remaining above 3.89. Notably, aside from the present study’s Source mean score (2.66), university cohorts (represented by solid lines) display trends similar to those observed in high school education samples (dashed lines). However, greater variability is evident in the Source and Certainty dimensions, particularly in Source, where the Mexican university cohort records the lowest score among university and secondary school samples, closely matching that of Taiwanese junior high students (2.60). Similarly, the Mexican sample’s Certainty mean score (3.55) surpasses only those of the USA elementary (3.38) and Taiwanese junior high (2.66) samples.

## 4. Discussion

### 4.1. Psychometric Properties of the Spanish SEB Questionnaire (RQ1)

This study examined evidence related to the psychometric properties of the Spanish version of the SEB Questionnaire in a sample of 791 students from a private university in northeastern Mexico. Both EFA and CFA supported a robust four-factor structure comprising Source, Certainty, Development, and Justification. Refinements resulted in a 22-item version that showed acceptable-to-good fit indices in CFA (CFI = 0.944, TLI = 0.936, RMSEA = 0.067, SRMR = 0.071), satisfactory internal consistency (Cronbach’s alpha = 0.77–0.87; CR = 0.80–0.92), and AVE values providing evidence consistent with convergent validity (AVE = 0.50–0.61).

Evidence regarding discriminant validity was generally adequate, although a notable conceptual overlap was observed between the Development and Justification factors (r = 0.780; AVE = 0.61, MSV = 0.61), suggesting that students who rely on evidence-based reasoning—through inquiry and experimentation—also tend to view scientific knowledge as tentative and subject to change. Certainty and Source correlate moderately (r = 0.685), reflecting the link between beliefs about how certain knowledge is and where it comes from (scientists, teachers or textbooks).

Prior research has reported internal-structure evidence consistent with a four-dimensional model. For example, Voitle et al. (2022) and Urhahne and Kremer (2023) validated 13- and 23-item models, respectively, with German secondary students and pre-service teachers, showing intercorrelation patterns similar to those observed in the present study. Likewise, Lin and Tsai (2017) reported comparable reliability and validity indices for a 21-item SEB model among Taiwanese high school students.

Findings from the IRT–GRM analysis indicated strong item discrimination across the epistemic-belief spectrum. Source items (1–4) were particularly effective for assessing medium-to-high levels of epistemic beliefs, whereas Certainty items (5–6, 8–11) provided more precise measurement at lower-to-middle levels of the construct. Taken together, the items functioned across a range of −2.58 to +2.91 on the latent trait continuum, indicating that the scale captures substantial variability within the targeted ability levels and is well suited for university student populations.

Items for Development (12–15, 17) and Justification (20–26) exhibited predominantly negative threshold values (−4.51 to −0.04) on the latent construct continuum. This pattern suggests that the items are relatively easy to endorse, which suggests that most students already view scientific knowledge as evolving and justified through empirical experimentation. Consequently, these items offer limited differentiation at higher trait levels, which may contribute to the conceptual overlap observed between both constructs. Although the same items show high discrimination parameters (>2.0 in most cases), their sensitivity is concentrated at lower to moderate levels of epistemic sophistication—an alignment that reflects the developmental stage for which the original instrument was designed (i.e., elementary school students), rather than the more advanced epistemic profiles expected in university populations.

The present study’s IRT analysis suggests that the Development and Justification items are particularly well-suited for students at the elementary and lower secondary levels (Mean scores ranging from 3.60 to 3.91, and 3.89 to 4.26, respectively). At these educational stages, science curricula typically begin to emphasize experimentation as a form of scientific evidence and introduce foundational laboratory practices. Conversely, among high school and university populations, these items exhibit a ceiling effect, with mean scores predominantly exceeding 4.00, indicating that most students have already internalized these aspects of scientific sophistication. While these findings corroborate the expected developmental progression within the sample, they also highlight the need for future research involving higher education cohorts to incorporate more complex and nuanced items to effectively capture the subtleties of epistemic beliefs within these dimensions.

Areepattamannil et al. (2020), using data from the PISA 2015 study, found no significant association between inquiry-based science instruction and epistemological beliefs, but did find a positive association with teacher-directed instruction. Based on the information provided by the authors, it can be inferred that PISA assesses epistemic beliefs through six SEB items—13, 15, and 17 for Development, and 21, 23, and 24 for Justification. In light of the findings of the present study, it is plausible that these items have a limited effect on the observed correlations, given their low threshold values and reduced discriminative power at higher levels of epistemic sophistication. By contrast, items related to Source and Certainty may be better suited to capture stronger associations with inquiry-based instruction, as they demonstrate greater measurement precision at more advanced stages of epistemic belief development, making them particularly appropriate for more academically experienced populations, such as high school students.

DIF analyses indicated generally small differences in item functioning across gender, academic semester, and faculty, consistent with measurement invariance assumptions and supporting the appropriateness of score comparisons across groups. It is important to note, however, that the underrepresentation of certain subgroups can influence DIF outcomes, highlighting the need for more balanced sampling in future applications. Additionally, the IRT analysis—encompassing both the GRM and DIF procedures—identified key items that could inform the development of a Spanish short version of the SEB Questionnaire.

**Figure 15:**
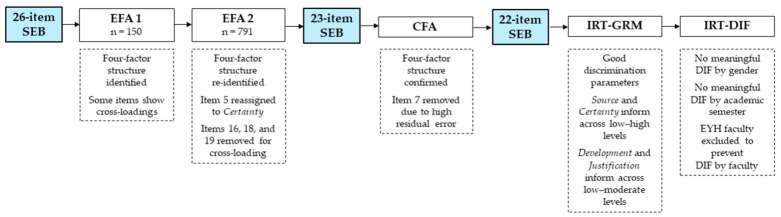
Analytical Framework for the Psychometric Evaluation of the SEB Questionnaire.

Figure 15 illustrates the practical contribution of each analytic procedure to the psychometric evaluation of the instrument, showing how they operate at different levels and degrees of precision. These complementary analyses provide a clearer understanding of the instrument’s functioning, underscoring the practical value of IRT-based approaches in supporting more accurate interpretation of the results.

### 4.2. Differences in Epistemic Beliefs by Gender and Academic Semester (RQ2)

Regarding group comparisons, female students consistently scored higher than male students across all epistemic belief dimensions, particularly in Certainty (M = 3.64 vs. 3.45), with a statistically significant difference (*p* = 0.0006). However, the effect size was small (r = 0.123). These patterns are consistent with prior research documenting gender differences in epistemic beliefs, as summarized in Table 17.

Moreover, female students exhibited significant increases in Source (*p* = 0.015–0.027) and Certainty (*p* = 0.043) across academic semesters, suggesting a pattern consistent with developmental progression in epistemic beliefs over time. In contrast, male students displayed more stable patterns, with no significant changes across semesters (see Figure 11 and Figure 12). Likewise, W. W. S. Lee and Chan (2015) found a positive correlation between Development and academic level among college students in Hong Kong, although they did not observe significant gender differences.

Particularly notable is the lower score and the lack of progression in Certainty among males (M = 3.45, SD = 0.81) compared to females (M = 3.64, SD = 0.84). This pattern may suggest that males tend to favor definite answers rather than conceiving knowledge in more graded terms. This female advantage aligns with the findings of Yang et al. (2014), whose eye-tracking study during science-text reading revealed significant gender differences in interaction with the Certainty dimension. Specifically, females demonstrated higher fluency in processing scientific narratives—a trend consistent with their more advanced epistemic performance, which may be associated with more efficient evaluation of scientific explanations.

Evidence from studies in Israel, Taiwan, and Finland using different epistemic belief instruments suggests a consistent gender pattern across diverse contexts. Female students have been found to be more evaluativist and less absolutist than males, to score higher in dimensions such as Source, Certainty, and Development, and to demonstrate a stronger understanding of scientific inquiry and knowledge construction (Tabak & Weinstock, 2008; Chiu et al., 2015; Yang et al., 2018; Lonka et al., 2021). Similar trends have been observed in Spanish samples using Schommer’s EQ, with female students showing higher scores and greater progression across academic levels (Cuéllar Fajardo & Martínez-Olmo, 2017; Alabau et al., 2020).

Taken together, these findings suggest that the gender differences observed in the present study may reflect a broader pattern rather than being specific to the present context. A similar trend is observed in large-scale assessments such as PISA 2018 and 2022, where girls have consistently outperformed boys in reading across participating countries (Organisation for Economic Co-operation and Development [OECD], 2023a, 2025). This advantage may be particularly meaningful, as greater reading proficiency is associated with increased engagement in internal argumentation processes, which facilitate epistemic cognition (Barzilai & Zohar, 2016).

Regarding students’ responses to instructional practices, the greater progression observed among female students—particularly in the Certainty dimension—may suggest that they begin questioning and revising their assumptions about knowledge earlier than male students. The relative stability among males may indicate that, in the absence of explicit epistemic interventions, they rely more heavily on direct instruction to modify their beliefs. In contrast, females appear more likely to challenge conventional approaches even when epistemic interventions are not explicitly designed.

From a sociocognitive perspective, these tendencies may reflect classroom interaction patterns and gendered approaches to knowledge. Jordan (2015) observed that females more frequently Request Help, whereas males tend to Deny Uncertainty. Similarly, females may engage in relational ways of knowing, efficiently integrating multiple perspectives, while males favor independent, answer-focused strategies (Clinchy, 2002). However, these patterns should be interpreted cautiously, as evidence regarding gender differences is still inconclusive.

### 4.3. Differences in Epistemic Beliefs by Faculty (RQ2): Interpretation and Implications

Regarding faculty differences, this study found only small effects (see Table 14). Even so, the SEB Questionnaire revealed subtle and significant variations among fields such as engineering (IYT), medicine (CS), and business (N). Contrary to prior research suggesting that students in natural sciences and engineering often view knowledge as more certain than those in the humanities and social sciences (Lonka et al., 2021), business students in this sample exhibited more absolutist beliefs, reflecting a tendency to view scientific knowledge as fixed, whereas engineering and health sciences students were more likely to see knowledge as evolving through inquiry and evidence.

These differences may be related to curricular emphases. Engineering and medicine programs typically involve empirical testing and problem-solving, whereas business curricula provide fewer opportunities to explore the provisional nature of scientific knowledge. These findings may suggest a potential need for business programs to integrate more explicit epistemic thinking, particularly given evidence that pseudoscientific endorsement is higher among business students than engineering students, signaling a possible gap in scientific education (Azuela et al., 2025).

While faculty differences may be partially attributed to curricular influences, a key question persists: if engineering and business are fundamentally distinct disciplines, why do differences in epistemic belief scores remain relatively modest? A more nuanced examination of these disciplinary patterns is necessary.

Some insight may be gained from the removal of item 7 during the CFA in the present study’s psychometric analysis. An item such as *“The most important part of doing science is coming up with the right answer”*, may introduce ambiguity due to the coexistence of multiple interpretive frameworks (Mason, 2016). This ambiguity likely stems from the item’s unspecified reference to “science,” which may evoke different disciplinary contexts. For example, some respondents may interpret it through empirical fields such as psychology, whereas others may consider more formal domains such as mathematics. Such variation echoes long-standing debates on the domain-generality versus domain-specificity of epistemic cognition (Hofer, 2016; Sandoval et al., 2016). Theoretical frameworks have similarly highlighted how epistemic beliefs are shaped within disciplinary practices and knowledge structures (Bromme et al., 2008; Elby et al., 2016; Hammer & Elby, 2002), and recent work continues to reinforce this multidimensional and context-sensitive perspective (DeGlopper & Stowe, 2024).

### 4.4. Contextualizing the Mexican Epistemic Profile Within International Data (RQ3)

As shown in Table 16, there is evidence that the SEB instrument has been applied across a wide range of educational levels, from elementary (Conley et al., 2004) and secondary (Peer, 2005) to university students (Urhahne & Kremer, 2023; Yang et al., 2014). Although the questionnaire demonstrates broad applicability, the absence of IRT analyses prevents the scoring from being refined, thereby limiting the precision and comparability of scores across groups.

A descriptive comparison offers preliminary insight into the epistemic profile of participants in the present study. A salient pattern is observed in the Mexican university cohort, particularly in the Source (M = 2.66, SD = 0.72) and Certainty (M = 3.55, SD = 0.83) dimensions. Despite their advanced academic level, Mexican students showed the lowest Source scores among the higher education samples reviewed, with levels comparable to those reported for Taiwanese junior high school students (M = 2.60; Cheng, 2018).

This disparity suggests that although these students recognize the procedural complexity inherent in scientific knowledge, they nonetheless retain a marked epistemic reliance on external authorities and textbooks as definitive sources of truth. Even though female students demonstrated a slightly higher overall mean Source score (M = 2.72, SD = 0.74) compared to males, their scores still indicate only limited autonomy in epistemic reasoning. Similarly, the moderate Certainty scores, which fall below those of secondary students from Namibia (M = 3.92; Shaakumeni, 2019) indicate a persistent inclination toward viewing scientific knowledge as absolute and immutable.

Collectively, these findings suggest an educational context in Mexico that may inadvertently reinforce a “received” view of knowledge, in which scientific truths are seen as external and fixed rather than as tentative constructs for active learner engagement. This aligns with Castañeda and Ortiz (2017), who, using Schommer’s EQ, found that first-semester psychology students in Mexico with strong, authority-dependent views of knowledge tended to perform worse academically.

In Mexico, sociocultural factors appear to support these source-dependent trajectories, as values of authority and hierarchy reinforce reliance on external sources of knowledge. For example, a phenomenological study of university students from northwest Mexico found that participants are often unaware of the dynamics in their interactions with professors, perceiving authority mainly in terms of power, respect, and recognition (Hernández Camarena et al., 2021).

Similarly, data from national studies and the World Values Survey—as reported by Moreno (2025)—indicate that the desire to make parents proud and seek their approval has remained strong, with around 90% agreement from 2000 to 2023. Mexican society continues to value parental authority and family ties, consistently ranking above the global average in these traditional expressions. Promoting more advanced epistemic beliefs therefore involves educational approaches that enable students to engage with scientific uncertainty and evidence in a more autonomous and evaluative manner.

### 4.5. Limitations and Directions for Future Research

Several limitations should be considered when interpreting these findings. First, self-report instruments are inherently vulnerable to response biases, which may affect the precision of the data. Second, the cross-sectional design prevents conclusions about the developmental trajectory of epistemic beliefs. Longitudinal work—ideally spanning elementary through postgraduate education—would clarify whether the gender-related patterns observed here persist across the educational lifespan and help identify the variables that shape them.

Third, although the overall sample size was adequate, some faculties—particularly Education and Humanities—were underrepresented, which may limit the generalizability of faculty-level comparisons. Additionally, the SEB Questionnaire was originally developed for elementary school students, and its application to a university population may partly account for the conceptual overlap observed between the Development and Justification dimensions. This suggests the need to refine certain constructs for use with older populations, both conceptually and psychometrically.

A further limitation concerns the contextual specificity of the measure. In the Mexican and broader Latin American context, students draw on a wider set of epistemic sources than those typically included in standard instruments. Beyond academic authorities such as textbooks, teachers, and scientists, tradition and family play a central role as culturally embedded sources of knowledge. At the same time, younger generations—particularly Gen Z—navigate an information environment shaped by digital media, social networks, online platforms, and emerging tools such as artificial intelligence. Not accounting for this expanded set of knowledge sources may limit the depth of the resulting findings.

Finally, questionnaires can serve as indicators of individuals’ epistemic beliefs but do not directly capture epistemic cognition (Sinatra, 2016). They primarily reflect how participants conceptualize and report their understanding of knowledge, making them indirect indicators of underlying epistemic processes. Future research would benefit from combining self-reports with complementary approaches that move beyond Likert-type scales, enabling the examination of epistemic cognition in more contextualized and process-oriented ways (Barzilai & Weinstock, 2015; Chinn et al., 2011).

Moving forward, it may be interesting to examine how individuals shift and activate different epistemic resources (Muis et al., 2006) depending on contextual and disciplinary demands—an approach aligned with theories of cognitive flexibility (Schommer-Aikins, 2011). Rather than treating epistemic beliefs as fixed traits, this perspective emphasizes the dynamic ways in which individuals adapt their reasoning to situational and disciplinary contexts. Advancing this line of work may require conceptualizing a broader framework that integrates epistemic beliefs with the emotional responses associated with uncertainty. In this regard, the term epistemic flexibility may offer a useful conceptual label for this emerging line of research, highlighting the context-sensitive regulation and adaptation of epistemic stances.

## 5. Conclusions

This study provides evidence consistent with a four-factor structure of the refined 22-item Spanish SEB and the reliability of scores in the present sample. EFA and CFA indicate a stable structure with adequate internal consistency, while IRT results show adequate to strong discrimination and coverage across the latent continuum. DIF analyses across gender, academic semesters, and faculties revealed generally small effect sizes, with limited evidence of differences in item functioning across groups, consistent with patterns expected under measurement invariance assumptions.

Female students tended to report higher epistemic belief scores and greater development across semesters, whereas differences across faculties were comparatively small. The Mexican cohort showed lower levels of epistemic autonomy, particularly in the Source and Certainty dimensions, compared to those reported in previous studies conducted in other countries, which may reflect contextual or educational influences that warrant further investigation.

Overall, these findings contribute evidence supporting the internal structure of the SEB and the interpretation of its scores in Spanish-speaking higher education contexts.

## Figures and Tables

**Table 1:** Participant distribution by gender across six academic faculties (*n* = 791).

Faculty	Male	Female	Total
AAYD	36 (4.6%)	95 (12.0%)	131 (16.6%)
CS	52 (6.6%)	145 (18.3%)	197 (24.9%)
DYCS	43 (5.4%)	40 (5.1%)	83 (10.5%)
EYH	17 (2.1%)	30 (3.8%)	47 (5.9%)
IYT	77 (9.7%)	34 (4.3%)	111 (14.0%)
N	130 (16.4%)	92 (11.6%)	222 (28.1%)
Total	355 (44.9%)	436 (55.1%)	791 (100.0%)

Note. AAYD = Art, Architecture, and Design; CS = Health Sciences; DYCS = Law and Social Sciences; EYH = Education and Humanities; IYT = Engineering and Technologies; N = Business.

**Table 2:** Rotated Factor Matrix of the 26-item SEB Questionnaire (*n* = 791).

Item	Factor			
	1	2	3	4
	Source	Certainty	Development	Justification
1	**0.714**	0.263	0.016	−0.037
2	**0.766**	0.126	−0.046	−0.198
3	**0.650**	0.309	−0.016	0.070
4	**0.737**	0.268	−0.086	−0.065
5	0.311	**0.579**	−0.074	0.040
6	0.168	**0.670**	0.113	0.029
7	0.021	**0.624**	−0.126	−0.067
8	0.106	**0.737**	0.157	0.160
9	0.351	**0.628**	0.045	−0.085
10	0.142	**0.786**	0.087	0.071
11	0.159	**0.702**	0.112	0.093
12	−0.121	0.093	**0.617**	0.292
13	−0.002	0.037	**0.781**	0.200
14	−0.055	0.048	**0.627**	0.339
15	0.052	0.040	**0.792**	0.304
16	−0.059	0.064	**0.685**	0.427
17	0.041	0.001	**0.731**	0.312
18	−0.072	0.024	0.467	**0.567**
19	−0.068	0.117	0.454	**0.619**
20	−0.077	0.046	0.356	**0.673**
21	−0.098	0.110	0.323	**0.724**
22	0.083	0.081	0.188	**0.705**
23	−0.016	−0.024	0.178	**0.729**
24	−0.077	−0.032	0.199	**0.692**
25	−0.056	0.088	0.223	**0.733**
26	−0.028	−0.041	0.183	**0.662**

Note. Extraction Method: Principal Component Analysis. Rotation Method: Varimax with Kaiser Normalization. Rotation converged in 7 iterations. All factor loadings are presented. Although loadings < 0.40 are reported, a threshold of ≥0.40 was applied for interpretative significance. The highest loading for each item is indicated in bold.

**Table 3:** Internal Consistency of the 23-item SEB Questionnaire.

Factor	Mean	SD	Number of Items	Cronbach’s *α*
Source	10.66	8.26	4	0.766
Certainty	24.06	5.66	7	0.827
Development	26.39	4.03	5	0.838
Justification	39.87	5.45	7	0.869
Total SEB	100.98	11.90	23	0.845

Note. Item 5 was reassigned to the Certainty subscale. Items 16, 18, and 19 were removed due to cross-loadings. After CFA, item 7 was excluded, leaving six items for Certainty (α = 0.827). The total 22-item SEB scale showed good reliability (α = 0.848).

**Table 4:** Latent Factor Variances (*ψ*) Summary.

Latent Factor	Variance (*ψ*)	Std. Error (SE)	*z*-Value
Source	0.580	0.035	13.819
Certainty	0.523	0.029	23.140
Development	0.675	0.032	16.322
Justification	0.483	0.030	19.204

Note. All factor variances were statistically significant (*p* < 0.001). According to conventional interpretation, values around 0.30–0.50 represent moderate variance, whereas values above 0.60 denote high variance, reflecting greater dispersion among participants on each latent construct.

**Table 5:** Composite Reliability, AVE, MSV and Inter-Factor Correlations (r).

Factor	CR	AVE	MSV	1	2	3	4
1. Source	0.80	0.50	0.47	**0.709**			
2. Certainty	0.86	0.52	0.47	0.685	**0.784**		
3. Development	0.90	0.65	0.61	−0.084	0.265	**0.804**	
4. Justification	0.92	0.61	0.61	−0.170	0.208	0.780	**0.784**

Note. CR: Composite Reliability, AVE: Average Variance Extracted, MSV: Maximum Shared Variance. The diagonal numbers in bold are the square root of the AVE values, which is the amount of variance a factor shares with its own items. The off-diagonal elements are the Standardized Correlations (r) between the factors, which is the variance they share with each other.

**Table 6:** Slope (a) and thresholds (b) parameters for the SEB Questionnaire.

Factor	Item	Discrimination	Thresholds			
		(a)	b_1_	b_2_	b_3_	b_4_
Source	1	1.74	−2.33	−0.39	1.34	2.21
	2	1.94	−1.34	0.36	1.87	2.91
	3	1.55	−2.41	−0.82	0.88	2.00
	4	2.22	−1.61	0.09	1.35	2.27
Certainty	5	1.32	−2.30	−0.67	0.73	1.90
	6	1.55	−2.33	−1.46	−0.35	0.72
	8	2.43	−2.58	−1.53	−0.74	−0.06
	9	1.73	−2.29	−0.82	0.55	1.56
	10	2.76	−2.16	−1.23	−0.31	0.50
	11	1.91	−2.35	−1.08	−0.17	0.75
Development	12	1.74	−3.09	−2.30	−1.16	−0.15
	13	2.87	−2.65	−2.01	−1.06	−0.04
	14	2.08	−3.47	−2.46	−1.48	−0.70
	15	4.30	−2.53	−2.04	−1.19	−0.38
	17	2.69	−2.86	−2.15	−1.12	−0.20
Justification	20	2.33	−3.59	−2.60	−1.39	−0.30
	21	3.14	−2.96	−2.46	−1.42	−0.70
	22	1.99	−3.33	−2.57	−1.28	−0.34
	23	2.22	−3.37	−2.71	−1.35	−0.31
	24	1.97	−3.86	−2.63	−1.36	−0.23
	25	2.43	−3.21	−2.46	−1.30	−0.30
	26	1.67	−4.51	−3.01	−1.50	−0.25

**Table 7:** Gender-Based DIF Analysis Using Likelihood Ratio χ^2^ Tests.

Likelihood Ratio Test (LRT)								Effect Size Indicators						β_12_
Item	ncat	*p*-Values			*df*			McFadden’s Pseudo-*R*^2^			Nagelkerke’s Pseudo-*R*^2^			
		χ^2^_12_	χ^2^_13_	χ^2^_23_	12	13	23	12	13	23	12	13	23	
1	5	0.139	0.315	0.728	1	2	1	0.001	0.001	<0.001	0.001	0.001	<0.001	0.003
2	5	0.143	0.250	0.426	1	2	1	0.001	0.001	<0.001	0.001	0.002	<0.001	0.007
3	5	0.406	0.693	0.833	1	2	1	<0.001	<0.001	<0.001	<0.001	<0.001	<0.001	0.003
4	5	**0.003**	**0.003**	0.120	1	2	1	0.004	0.006	0.001	0.004	0.005	0.001	0.019
5	5	0.270	0.531	0.823	1	2	1	<0.001	<0.001	<0.001	0.001	0.001	<0.001	0.007
6	5	**0.003**	**0.012**	0.738	1	2	1	0.004	0.004	<0.001	0.006	0.007	<0.001	<0.001
8	5	0.626	0.866	0.824	1	2	1	<0.001	<0.001	<0.001	<0.001	<0.001	<0.001	<0.001
9	5	0.172	0.177	0.206	1	2	1	<0.001	0.002	<0.001	0.001	0.002	0.001	0.006
10	5	0.783	0.825	0.579	1	2	1	<0.001	<0.001	<0.001	<0.001	<0.001	<0.001	<0.001
11	5	0.072	0.125	0.339	1	2	1	0.001	0.002	<0.001	0.002	0.002	<0.001	0.003
12	5	0.914	0.846	0.569	1	2	1	<0.001	<0.001	<0.001	<0.001	<0.001	<0.001	<0.001
13	4	0.988	0.417	0.186	1	2	1	<0.001	0.001	0.001	<0.001	<0.001	<0.001	<0.001
14	4	0.413	0.272	0.164	1	2	1	<0.001	0.002	0.001	<0.001	0.002	0.002	0.002
15	3	0.872	0.950	0.782	1	2	1	<0.001	<0.001	<0.001	<0.001	<0.001	<0.001	<0.001
17	4	0.501	0.307	0.167	1	2	1	<0.001	0.001	0.001	<0.001	0.001	0.001	0.001
20	4	0.328	0.598	0.787	1	2	1	<0.001	<0.001	<0.001	<0.001	<0.001	<0.001	0.002
21	3	0.169	**<0.001**	**<0.001**	1	2	1	0.002	0.018	0.016	0.001	0.015	0.014	0.003
22	4	0.647	0.439	0.231	1	2	1	<0.001	0.001	<0.001	<0.001	0.001	0.001	0.002
23	4	0.584	0.838	0.818	1	2	1	<0.001	<0.001	<0.001	<0.001	<0.001	<0.001	0.002
24	4	0.551	0.675	0.511	1	2	1	<0.001	<0.001	<0.001	<0.001	<0.001	<0.001	0.003
25	3	0.401	0.315	0.205	1	2	1	<0.001	0.002	0.001	<0.001	0.001	0.001	0.002
26	4	0.946	0.905	0.658	1	2	1	<0.001	<0.001	<0.001	<0.001	<0.001	<0.001	<0.001

Note. ncat = number of response categories; *p*-values reflect likelihood ratio χ^2^ comparisons between nested logistic regression models: χ^2^_12_ (uniform DIF), χ^2^_13_ (non-uniform DIF), and χ^2^_23_ (global DIF). The corresponding *df* values are listed for each test. Effect size indicators include changes in McFadden’s, Nagelkerke’s pseudo-*R*^2^, and β_12_ (the absolute proportional change in the ability slope between Models 1 and 2). *p* < 0.05, effect size estimates > 0.02, and β_12_ > 0.03 are presented in bold.

**Table 8:** Mann–Whitney U test comparing gender on SEB Dimensions (M = 355, F = 436).

Dimension	U	Z	Adjusted *p*-Value	Effect Size (r)
Source	69,362	−2.529	0.011 *	0.090 (negligible)
Certainty	66,376	−3.453	0.0006 ***	0.123 (small)
Development	65,518	−3.146	0.002 **	0.112 (small)
Justification	67,400	−3.156	0.002 **	0.112 (small)
Total SEB	63,782	−4.260	0.00002 ***	0.152 (small)

Note. The table shows the Mann–Whitney U test statistics (U), standardized test statistics (Z), adjusted *p*-values, and effect sizes (r) for gender comparisons across four dimensions of SEB. Significance levels are denoted as follows: * *p* < 0.05, ** *p* < 0.01, *** *p* < 0.001.

**Table 9:** Descriptive statistics of SEB scores by gender.

Dimension	Mean (SD)				
	Source	Certainty	Development	Justification	Total SEB
Female (*n* = 436)	2.72 (0.74)	3.64 (0.84)	4.42 (0.69)	4.48 (0.61)	3.92 (0.46)
Male (*n* = 355)	2.59 (0.69)	3.45 (0.81)	4.31 (0.67)	4.37 (0.60)	3.78 (0.45)
Total (*n* = 791)	2.66 (0.72)	3.55 (0.83)	4.37 (0.68)	4.43 (0.61)	3.86 (0.46)

**Table 10:** Kruskal–Wallis test for SEB scores across academic semesters (K = 4), stratified by gender.

Gender	*n*	Dimension	H	*df*	*p*-Value	Effect Size (η^2^_H)
Male (M)	286	Source	0.087	3	0.993	0.000 (no effect)
		Certainty	2.682	3	0.443	0.000 (no effect)
		Development	6.138	3	0.105	0.011 (small)
		Justification	6.977	3	0.073	0.014 (small)
		Total SEB	6.643	3	0.084	0.013 (small)
Female (F)	383	Source	13.04	3	0.005 **	0.026 (small)
		Certainty	9.77	3	0.021 *	0.018 (small)
		Development	0.84	3	0.840	0.000 (no effect)
		Justification	2.52	3	0.472	0.000 (no effect)
		Total SEB	10.23	3	0.017 *	0.019 (small)

* *p* < 0.05, ** *p* < 0.01.

**Table 11:** Pairwise Mann–Whitney U tests assessed differences in SEB scores across academic semesters for the female group (*n* = 383).

Dimension	Semester Groups (n1–n2)	U	Z	*p*-Value (Adjusted)	Effect Size (r)
Source	2–4 (85–144)	4752	−2.846	0.027 *	0.188 (small)
	2–6 (85–97)	3068	−2.999	0.016 *	0.222 (small)
	2–8 (85–57)	1702	−3.024	0.015 *	0.254 (small)
Certainty	2–4 (85–144)	4947	−2.428	0.091	0.160 (small)
	2–6 (85–97)	3245	−2.480	0.079	0.184 (small)
	2–8 (85–57)	1776	−2.694	0.043 *	0.226 (small)
Total SEB	2–4 (85–144)	4729	−2.874	0.024 *	0.190 (small)
	2–6 (85–97)	3217	−2.555	0.064	0.189 (small)
	2–8 (85–57)	1846	−2.403	0.098	0.202 (small)

* *p* < 0.05.

**Table 12:** Descriptive statistics of SEB scores by academic semester for the female group (*n* = 383).

Semester	Mean (SD)				
	Source	Certainty	Development	Justification	Total SEB
2 (*n* = 85)	2.45 (0.63)	3.36 (0.92)	4.36 (0.72)	4.41 (0.65)	3.76 (0.50)
4 (*n* = 144)	2.74 (0.73)	3.69 (0.76)	4.48 (0.58)	4.53 (0.54)	3.96 (0.44)
6 (*n* = 97)	2.79 (0.76)	3.68 (0.89)	4.40 (0.72)	4.54 (0.61)	3.96 (0.43)
8 (*n* = 57)	2.85 (0.78)	3.80 (0.71)	4.43 (0.74)	4.51 (0.52)	4.00 (0.47)

**Table 13:** Kruskal–Wallis test for SEB scores across faculties (K = 5, *n* = 744).

Dimension	H	*df*	*p*-Value	Effect Size (η^2^_H)
Source	0.087	4	0.063	0.007 (small)
Certainty	2.682	4	0.053	0.007 (small)
Development	6.138	4	0.00004 ***	0.029 (small)
Justification	6.977	4	0.004 **	0.015 (small)
Total SEB	6.643	4	0.0013 **	0.019 (small)

** *p* < 0.01, *** *p* < 0.001.

**Table 14:** Pairwise Mann–Whitney U tests assessed differences in SEB scores across faculties.

Dimension	Faculty Groups (n1–n2)	U	Z	*p*-Value (Adjusted)	Effect Size (r)
Source	AAYD−IYT (131–111)	5745	−2.832	0.046 *	0.182 (small)
Certainty	IYT−N (111–222)	9886	−2.947	0.032 *	0.162 (small)
Development	CS−N (197–222)	15,812	−4.984	0.00001 ***	0.244 (small)
Justification	CS−N (197–222)	17,132	−3.866	0.001 **	0.189 (small)
Total SEB	CS−N (197–222)	16,853	−4.055	0.0005 ***	0.198 (small)

* *p* < 0.05, ** *p* < 0.01, *** *p* < 0.001.

**Table 15:** Descriptive statistics of SEB scores by faculty (*n* = 744).

Faculty	Mean (SD)				
	Source	Certainty	Development	Justification	Total SEB
AAYD	2.80 (0.75)	3.60 (0.85)	4.33 (0.75)	4.41 (0.70)	3.88 (0.45)
CS	2.61 (0.67)	3.57 (0.77)	4.54 (0.58)	4.56 (0.52)	3.93 (0.42)
DYCS	2.69 (0.73)	3.50 (0.94)	4.33 (0.77)	4.41 (0.69)	3.83 (0.48)
IYT	2.55 (0.75)	3.68 (0.85)	4.40 (0.60)	4.46 (0.55)	3.89 (0.46)
N	2.64 (0.75)	3.42 (0.82)	4.24 (0.68)	4.33 (0.61)	3.76 (0.47)

**Table 16:** Exemplary empirical studies applying the SEB Questionnaire.

Authors	Country	Sample	Items	Dimension Mean Scores (SD)			
				S	C	D	J
Higher Education							
Present Study	Mexico	791 (University)	22	2.66 (0.72)	3.55 (0.83)	4.37 (0.68)	4.43 (0.61)
Urhahne and Kremer (2023)	Germany	196 (Pre-service teachers)	23	3.18 (0.75)	3.75 (0.75)	3.90 (0.75)	4.01 (0.75)
Yang et al. (2014)	Taiwan	25 (University)	26	3.39 (0.56)	3.70 (0.61)	4.32 (0.60)	4.22 (0.54)
Secondary and High School							
Shaakumeni (2019)	Namibia	944 (11–12th grade)	22	3.36 (0.89)	3.92 (0.78)	4.14 (0.62)	4.26 (0.56)
Lin and Tsai (2017)	Taiwan	600 (High school)	21	3.25 (0.75)	3.81 (0.68)	4.20 (0.61)	4.10 (0.61)
Voitle et al. (2022)	Germany	105 (10th grade)	13	3.31 (0.75)	4.00 (0.66)	4.21 (0.51)	4.13 (0.48)
Elementary and Lower Secondary							
Cheng (2018)	Taiwan	267 (Jr. High school)	17	2.60 (0.99)	2.66 (0.97)	3.81 (0.94)	3.89 (0.88)
Peer (2005)	Singapore	104 (8th grade)	25	3.93 (0.58)	4.04 (0.50)	3.91 (0.58)	4.10 (0.53)
Özkan and Tekkaya (2011)	Turkey	1230 (7th grade)	26	3.28 (0.64)		3.60 (0.61)	3.99 (0.62)
Conley et al. (2004)	USA	187 (5th grade)	26	3.68 (0.80)	3.38 (0.83)	3.90 (0.54)	4.26 (0.43)

Note. S = Source, C = Certainty, D = Development, J = Justification. Urhahne and Kremer (2023) scores in Natural Sciences for Source and Certainty were reversed to align with the directionality of other studies. Similarly, Yang et al. (2014) scores for Source and Certainty were reversed for consistency. Scores from Voitle et al. (2022) and Peer (2005) were converted from 7-point and 6-point scales, respectively, to a 5-point Likert scale. Özkan and Tekkaya (2011) utilized a 3-factor instrument, with one factor combining Source and Certainty. For Conley et al. (2004), post-intervention means were reported.

**Table 17:** SEB studies reporting higher female (F) scores compared to male (M) students.

Authors	Country	Sample	Dimension Mean Scores (M/F)				Findings
			S	C	D	J	
Present Study	Mexico	791 (University) (M/F = 44.9/55.1%)	2.59/2.72 * (0.69/0.74)	3.45/3.64 *** (0.81/0.84)	4.31/4.42 ** (0.67/0.69)	4.37/4.48 ** (0.60/0.61)	Sig. Diff. 4 Factors
Shaakumeni (2019)	Namibia	944 (11–12th grade) (M/F = 45/55%)	3.26/3.44 ** (0.89/0.89)	3.92/3.90 (0.80/0.77)	4.13/4.14 (0.62/0.61)	4.24/4.27 (0.57/0.55)	Sig. Diff. Source
Yang et al. (2014)	Taiwan	25 (University) (M/F = 52/48%)	-	3.50/3.95 ***	-	-	Sig. Diff. Certainty
Özkan and Tekkaya (2011)	Turkey	1230 (7th grade) (M/F = 51.8/48.2%)	3.30/3.26 (0.63/0.64)		3.59/3.60 (0.62/0.59)	3.89/4.09** (0.68/0.56)	Sig. Diff. Justification

Note. S = Source, C = Certainty, D = Development, J = Justification. For Yang et al. (2014), scores are reported by gender only for Certainty and were reversed for consistency. Özkan and Tekkaya (2011) used a 3-factor instrument, with one factor combining both Source and Certainty. * *p* < 0.05, ** *p* < 0.01, *** *p* < 0.001.

**Figure A1:**
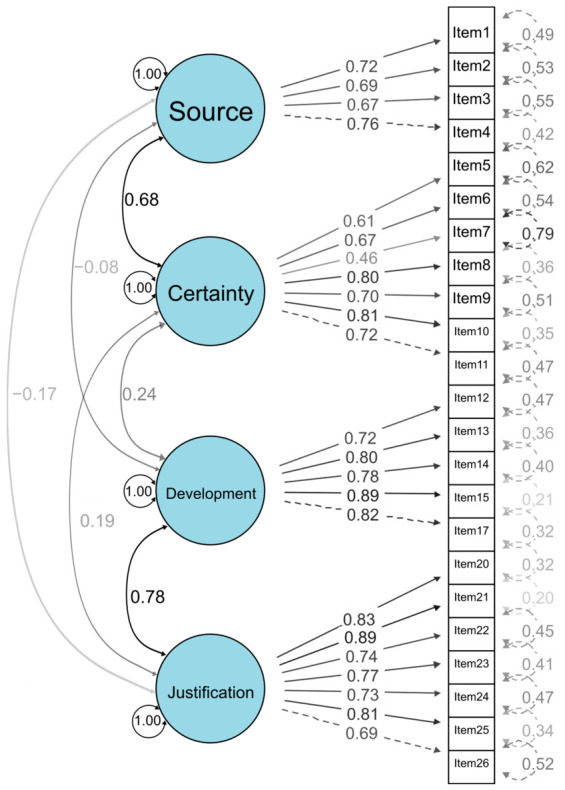
Confirmatory Factor Analysis (CFA) model of the 23-item SEB Questionnaire.

## Data Availability

Data are contained within the article.

## Abbreviations

The following abbreviations are used in this manuscript:

AAYD	Faculty of Art, Architecture, and Design
AVE	Average Variance Extracted
CFA	Confirmatory Factor Analysis
CFI	Comparative Fit Index
CR	Composite Reliability
CS	Faculty of Health Sciences
DIF	Differential Item Functioning
DYCS	Faculty of Law and Social Sciences
EFA	Exploratory Factor Analysis
EQ	Epistemological Beliefs Questionnaire
EYH	Faculty of Education and Humanities
GRM	Graded Response Model
ICCs	Item Characteristic Curves
IICs	Item Information Curves
IRT	Item Response Theory
IYT	Faculty of Engineering and Technologies
KMO	Kaiser–Meyer–Olkin test
LRT	Likelihood Ratio Test
MSV	Maximum Shared Variance
N	Faculty of Business
OECD	Organisation for Economic Co-operation and Development
PISA	Programme for International Student Assessment
RMSEA	Root Mean Square Error of Approximation
RQ	Research Question
SEB	Scientific Epistemic Beliefs Questionnaire
SRMR	Standardized Root Mean Square Residual
TCCs	Test Characteristic Curves
TLI	Tucker–Lewis Index
WLSMV	Weighted Least Squares Mean and Variance

## Appendix A

### Spanish Version of the Scientific Epistemic Beliefs (SEB) Questionnaire

**Fuente**

1. Todo el mundo debe creer lo que dicen los científicos.
2. En cuestiones científicas, hay que creer lo que dicen los libros de ciencia sobre las cosas.
3. Todo lo que dice el profesor o la profesora en la clase de ciencias es cierto.
4. Si lees algo en un libro de ciencias, puedes estar seguro de que es verdad.
5. Sólo los científicos saben con seguridad qué es lo cierto en la ciencia.

**Certeza**

6. Todas las preguntas científicas tienen solo una respuesta correcta.
7. La parte más importante de hacer ciencia es encontrar la respuesta correcta.
8. Los científicos saben prácticamente todo sobre la ciencia; no hay mucho más que saber.
9. El conocimiento científico siempre es cierto.
10. Una vez que los científicos obtienen el resultado de un experimento, esa es la única respuesta.
11. Los científicos siempre están de acuerdo sobre lo que es verdad en la ciencia.

**Desarrollo**

12. Algunas ideas de la ciencia actual son diferentes de lo que solían pensar los científicos antes.
13. Las ideas de los libros de ciencias a veces cambian.
14. Hay algunas preguntas que ni siquiera los científicos pueden responder.
15. Las ideas en la ciencia a veces cambian.
16. Nuevos descubrimientos pueden cambiar lo que los científicos creen que es verdad.
17. A veces los científicos cambian de opinión sobre lo que es verdad en la ciencia.

**Justificación**

18. Las ideas sobre experimentos científicos surgen de la curiosidad y el pensamiento sobre cómo funcionan las cosas.
19. En ciencia, puede haber más de una forma en que los científicos pongan a prueba sus ideas.
20. Una parte importante de la ciencia es hacer experimentos para generar nuevas ideas sobre cómo funcionan las cosas.
21. Es bueno probar experimentos más de una vez para asegurarse de sus hallazgos.
22. Las buenas ideas científicas pueden surgir de cualquiera, no sólo de los científicos.
23. Una buena manera de saber si algo es cierto es hacer un experimento.
24. Las buenas respuestas se basan en evidencias obtenidas de muchos experimentos diferentes.
25. Las ideas científicas pueden surgir de tus propias preguntas y experimentos.
26. Es bueno tener una idea antes de comenzar un experimento.

## Appendix B

### Exploratory Factor Analysis on a Pilot Sample of 150 University Students

**Table A1:** Rotated Factor Matrix of the 26-item SEB Questionnaire (*n* = 150).

Item	Factor			
	1	2	3	4
	Source	Certainty	Development	Justification
1	**0.715**	0.146	−0.204	0.113
2	**0.773**	0.180	−0.154	−0.059
3	**0.549**	0.492	0.001	0.083
4	**0.662**	0.348	0.054	−0.194
5	**0.607**	0.317	0.184	−0.109
6	0.205	**0.689**	0.110	−0.018
7	0.284	**0.608**	−0.075	0.012
8	0.142	**0.693**	0.108	0.231
9	0.431	**0.693**	0.014	0.039
10	0.140	**0.849**	0.108	0.126
11	0.044	**0.782**	0.131	0.152
12	−0.003	−0.009	**0.599**	0.294
13	−0.033	0.110	**0.743**	0.302
14	0.052	0.127	**0.596**	0.405
15	−0.142	0.228	**0.660**	0.405
16	−0.140	0.182	0.516	**0.609**
17	−0.066	0.021	**0.734**	0.316
18	−0.021	0.039	0.382	**0.608**
19	0.147	0.033	0.256	**0.704**
20	0.103	0.009	0.254	**0.786**
21	−0.014	0.135	0.222	**0.753**
22	−0.031	0.126	0.093	**0.711**
23	−0.217	0.182	0.062	**0.730**
24	−0.010	−0.041	0.212	**0.717**
25	−0.084	0.018	0.338	**0.735**
26	−0.030	0.091	0.274	**0.656**

Note. Extraction Method: Principal Component Analysis. Rotation Method: Varimax with Kaiser Normalization. Rotation converged in 7 iterations. All factor loadings are presented. Although loadings <0.40 are reported, a threshold of ≥0.40 was applied for interpretative significance. The highest loading for each item is indicated in bold.

## Appendix C

### Confirmatory Factor Analysis Model of the 23-Item SEB Questionnaire

Based on the exploratory factor analysis (EFA) conducted with the main sample (*n* = 791), items 16, 18, and 19 were removed from the original 26-item SEB Questionnaire, resulting in a 23-item version. Subsequently, the confirmatory factor analysis (CFA) indicated inadequate psychometric performance for item 7, which was therefore removed, yielding a final 22-item scale. Figure A1 presents the CFA results for the 23-item model prior to this final adjustment.

## Appendix D

### Appendix D.1. Academic Semester–Based Differential Item Functioning (DIF) Analysis Using Likelihood Ratio Tests (LRT)

**Table A2:** Semester-Based DIF Analysis Using Likelihood Ratio χ^2^ Tests.

Likelihood Ratio Test (LRT)								Effect Size Indicators						β_12_
Item	ncat	*p*-Values			*df*			McFadden’s Pseudo-*R*^2^			Nagelkerke’s Pseudo-*R*^2^			
		χ^2^_12_	χ^2^_13_	χ^2^_23_	12	13	23	12	13	23	12	13	23	
1	4	0.496	0.553	0.468	3	6	3	0.002	0.003	0.002	0.002	0.004	0.002	0.003
2	4	0.833	0.749	0.459	3	6	3	<0.001	0.002	0.002	<0.001	0.003	0.002	<0.001
3	5	0.262	0.583	0.872	3	6	3	0.002	0.003	<0.001	0.003	0.003	<0.001	<0.001
4	4	0.349	0.427	0.445	3	6	3	0.002	0.004	0.002	0.002	0.003	0.001	0.008
5	4	0.373	0.246	0.189	3	6	3	0.002	0.005	0.003	0.003	0.008	0.005	0.005
6	4	0.215	0.182	0.223	3	6	3	0.003	0.005	0.002	0.004	0.008	0.004	0.015
8	3	0.922	0.976	0.863	3	6	3	<0.001	<0.001	<0.001	<0.001	0.001	<0.001	0.003
9	4	0.138	0.204	0.396	3	6	3	0.003	0.005	0.002	0.005	0.007	0.003	0.014
10	4	0.344	0.367	0.362	3	6	3	0.002	0.004	0.002	0.001	0.003	0.001	0.002
11	4	0.061	0.193	0.732	3	6	3	0.004	0.005	<0.001	0.005	0.006	<0.001	0.005
12	3	0.893	0.927	0.728	3	6	3	<0.001	0.001	0.001	<0.001	0.002	0.001	<0.001
13	3	**0.001**	**<0.001**	**0.011**	3	6	3	0.012	0.020	0.008	0.009	0.015	0.006	0.012
14	2	0.349	0.699	0.909	3	6	3	0.004	0.005	<0.001	0.004	0.005	<0.001	0.006
15	2	0.975	0.965	0.755	3	6	3	<0.001	0.002	0.001	<0.001	0.001	<0.001	0.001
17	3	0.269	0.637	0.948	3	6	3	0.003	0.003	<0.001	0.003	0.003	<0.001	0.013
20	2	0.094	0.312	0.874	3	6	3	0.007	0.008	<0.001	0.007	0.008	<0.001	0.027
21	3	0.349	0.706	0.922	3	6	3	0.003	0.004	<0.001	0.003	0.003	<0.001	0.006
22	3	0.205	0.553	0.952	3	6	3	0.004	0.004	<0.001	0.004	0.004	<0.001	0.008
23	3	0.345	0.401	0.411	3	6	3	0.003	0.005	0.002	0.003	0.005	0.002	0.016
24	3	0.723	0.636	0.396	3	6	3	0.001	0.003	0.002	0.001	0.004	0.003	0.001
25	3	0.116	0.286	0.684	3	6	3	0.005	0.006	0.001	0.004	0.005	0.001	0.003
26	3	**0.014**	**0.028**	0.321	3	6	3	0.008	0.011	0.003	0.011	0.014	0.004	0.028

Note. ncat = number of response categories; *p*-values reflect likelihood ratio χ^2^ comparisons between nested logistic regression models: χ^2^_12_ (uniform DIF), χ^2^_13_ (non-uniform DIF), and χ^2^_23_ (global DIF). The corresponding *df* values are listed for each test. Effect size indicators include changes in McFadden’s, Nagelkerke’s pseudo-*R*^2^, and β_12_ (the absolute proportional change in the ability slope between Models 1 and 2). *p* < 0.05, effect size estimates > 0.02, and β_12_ > 0.03 are presented in bold.

### Appendix D.2. Faculty-Based Differential Item Functioning (DIF) Analysis Using Likelihood Ratio Tests (LRT)

**Table A3:** Faculty-Based DIF Analysis Using Likelihood Ratio χ^2^ Tests.

Likelihood Ratio Test (LRT)								Effect Size Indicators						β_12_
Item	ncat	*p*-Values			*df*			McFadden’s Pseudo-*R*^2^			Nagelkerke’s Pseudo-*R*^2^			
		χ^2^_12_	χ^2^_13_	χ^2^_23_	12	13	23	12	13	23	12	13	23	
1	2	0.506	0.882	0.976	5	10	5	0.004	0.005	<0.001	0.004	0.005	0.001	0.010
2	2	0.171	0.196	0.327	5	10	5	0.007	0.013	0.006	0.007	0.013	0.005	0.012
3	4	**<0.001**	**<0.001**	0.142	5	10	5	0.013	0.017	0.004	0.016	**0.022**	0.005	0.029
4	3	0.382	0.615	0.722	5	10	5	0.003	0.005	0.002	0.003	0.005	0.002	0.010
5	4	0.697	0.643	0.436	5	10	5	0.002	0.004	0.002	0.003	0.007	0.004	0.010
6	3	0.736	0.278	0.096	5	10	5	0.002	0.007	0.005	0.002	0.010	0.008	0.005
8	3	0.661	0.645	0.469	5	10	5	0.002	0.005	0.003	0.002	0.005	0.003	0.004
9	4	0.773	0.964	0.958	5	10	5	0.001	0.002	<0.001	0.002	0.003	0.001	0.007
10	3	0.616	0.318	0.158	5	10	5	0.002	0.007	0.005	0.002	0.005	0.003	0.003
11	3	0.087	0.305	0.837	5	10	5	0.006	0.007	0.001	0.007	0.008	0.001	0.007
12	3	0.132	0.406	0.859	5	10	5	0.005	0.007	0.001	0.007	0.008	0.002	0.010
13	2	0.295	0.191	0.186	5	10	5	0.006	0.012	0.007	0.004	0.009	0.005	0.002
14	2	0.161	0.536	0.960	5	10	5	0.008	0.009	0.001	0.009	0.010	0.001	**0.036**
15	3	**<0.001**	**<0.001**	0.042	5	10	5	0.017	**0.025**	0.008	0.010	0.014	0.005	**0.077**
17	3	0.928	0.983	0.907	5	10	5	<0.001	0.002	0.001	0.001	0.002	0.001	<0.001
20	2	0.766	0.700	0.454	5	10	5	0.002	0.007	0.004	0.002	0.007	0.004	0.012
21	2	0.591	0.334	0.180	5	10	5	0.004	0.012	0.008	0.003	0.010	0.007	<0.001
22	3	0.382	0.805	0.975	5	10	5	0.004	0.004	0.001	0.004	0.005	0.001	0.009
23	3	0.268	0.493	0.700	5	10	5	0.004	0.006	0.002	0.005	0.007	0.002	0.016
24	3	**0.018**	**<0.001**	**0.003**	5	10	5	0.009	**0.021**	0.012	0.010	**0.023**	0.013	**0.033**
25	3	0.405	0.313	0.260	5	10	5	0.003	0.008	0.004	0.003	0.007	0.004	0.006
26	3	0.366	0.584	0.695	5	10	5	0.004	0.006	0.002	0.005	0.007	0.003	0.003

Note. ncat = number of response categories; *p*-values reflect likelihood ratio χ^2^ comparisons between nested logistic regression models: χ^2^_12_ (uniform DIF), χ^2^_13_ (non-uniform DIF), and χ^2^_23_ (global DIF). The corresponding *df* values are listed for each test. Effect size indicators include changes in McFadden’s, Nagelkerke’s pseudo-*R*^2^, and β_12_ (the absolute proportional change in the ability slope between Models 1 and 2). *p* < 0.05, effect size estimates > 0.02, and β_12_ > 0.03 are presented in bold.
